# Pre-breeding efforts through introgression of pre-harvest sprouting resistance genes from wild *Vigna radiata* var. *sublobata* into cultivated mungbean

**DOI:** 10.3389/fpls.2025.1689852

**Published:** 2025-10-23

**Authors:** Deepak Khanderao Patil, Sachin Laxman Dhare, Vishnu Kishanrao Gite, Hirakant Vishwasrao Kalpande, Niranjan Ravindra Thakur, Aditya Pratap, Khizer Samad Baig, Rakesh Dnyandeo Ahire

**Affiliations:** ^1^ Department of Genetics and Plant Breeding, Agriculture Research Station, Badnapur, Jalna, MS, India; ^2^ Department of Genetics and Plant Breeding, Vasantrao Naik Marathwada Krishi Vidyapeeth, Parbhani, MS, India; ^3^ Crop Improvement Division, Indian Council of Agricultural Research (ICAR)-Indian Institute of Pulses Research, Kanpur, UP, India; ^4^ Department of Agriculture Extension, Vasantrao Naik Marathwada Krishi Vidyapeeth, Parbhani, MS, India

**Keywords:** fresh seed germination (FSG), introgression breeding, pre-breeding, pre-harvest sprouting (PHS), seed dormancy, *Vigna radiata* var. *sublobata*, wild mungbean

## Abstract

**Introduction:**

Pre-harvest sprouting (PHS), triggered by heavy, prolonged rainfall and high humidity during the pod ripening stage, is a significant constraint on mungbean production, causing severe losses in yield and quality. This results in poor seed quality and a reduced market value.

**Methods:**

This study, conducted at the Agriculture Research Station, Badnapur, Maharashtra, aimed to develop PHS-resistant mungbean lines through introgression of resistance genes from the wild species, *Vigna radiata* var. *sublobata*. Seven PHS-tolerant inter-specific derivatives were identified from a cross between the elite cultivar BM-4 and the wild accession BWM-29. These derivatives were further crossed with six locally adapted varieties from different agroclimatic regions, producing 42 cross combinations. Parental lines and their crosses were evaluated for PHS incidence and fresh seed germination (FSG) under control conditions.

**Results and discussion:**

Wide genetic variation was observed, for PHS ranging from 3.55% to 98.86% and FSG from 4.76% to 96.17%. Four crosses exhibited less than 10% PHS, indicating strong tolerance. Correlation analysis revealed a significant positive correlation between the FSG and PHS. Genetic analysis indicated that PHS is controlled by a single gene, with dormancy dominant over non-dormant, representing the major finding of this research. Principal component analysis revealed that PHS and FSG were the main contributors to the observed variability. Based on PHS tolerance, crosses were categorized as tolerant, moderately tolerant, moderately susceptible, and susceptible groups. The identified PHS-tolerant crosses and male parents will be valuable resources for mungbean breeding. This study highlights the importance of utilizing wild genetic resources and inter-specific hybridization to enhance PHS tolerance in the mungbean providing a robust foundation for breeding resilient, high yielding climate-adapted cultivars.

## 1 Introduction

Pulses are essential components of the global food system and serve as a primary and affordable source of dietary proteins, particularly in vegetarian populations. Their protein content ranges between 20 and 30%, which is approximately two–three times higher than that of cereals (Cardon-Thomas et al., 2017; Singh et al., 2021; Kadam et al., 2023). In addition to proteins, pulses are rich in complex carbohydrates, dietary fiber, vitamins, and minerals, making them valuable dietary components for addressing protein-energy malnutrition and micronutrient deficiencies. Among these, mungbean (*Vigna radiata* L. Wilczek) has gained global attention owing to its nutritional quality and its agronomic benefits. Its seeds contain approximately 15–30% protein (Dhare et al., 2024) and 53-67% carbohydrates (Anwar et al., 2007; Dahiya et al., 2015). Mungbean is also rich in essential amino acids, such as lysine, arginine, histidine, and tryptophan (Jayamani et al., 2015). Mungbean can serve as a supplement to cereal-based diets by supplying lysine, an essential amino acid that is deficient in cereals, thereby improving overall protein quality (Dhare et al., 2025). When sprouted, they become a source of antioxidants that promote gut health and may help prevent cardiovascular diseases and certain cancers (Nair et al., 2013; Ganesan and Xu, 2018). Agronomically, mungbean is well adapted to diverse environments, with rapid growth, nitrogen fixation ability, and tolerance to heat, drought, and salinity (Mishra et al., 2022; Mogotsi, 2006). These traits make it suitable for various cropping systems, particularly cereal-based systems. Mungbean is widely cultivated in South, East, and Southeast Asia and is expanding into new regions such as Australia, the United States, and Africa (Hou et al., 2019; Somta et al., 2022). India stands as the leading producer and consumer of mungbean, accounting for more than 70% of the world’s production (Kumari and Malik, 2024). During the 2024–2025 rainy season, mungbean was grown on 3.30 million hectares across the country, yielding 1.38 million tons with an average productivity of 418kg per hectare (https://www.indiastat.com/).

Evolutionarily, many plant species, including mungbean, have evolved seed dormancy as an adaptive trait (Jia et al., 2024). This adaptive trait allows seed dispersal, prevents germination under unfavorable conditions, and improves survival rates in diverse environments (Koornneef et al., 2002; Venable, 2007). Seed dormancy is attributed to morphological, physiological, morpho-physiological, and physical properties as well as a combination of physical and physiological properties (Baskin and Baskin, 2004). In legumes such as mungbean, hard-seededness causes physical dormancy and impedes water absorption, complicating milling (Paul et al., 2025). Through domestication, humans select crops with desirable cultivation traits, leading to genetic differences from their wild ancestors (Thakur et al., 2024). Some noteworthy examples include larger/bold seed size, uniform germination, reduced seed shattering, synchronous maturity, lower phenol or tannin content, and changes in seed coat color. While these traits enhance agricultural performance, they can inadvertently increase mungbean susceptibility to pre-harvest sprouting (PHS) (Lamichaney et al., 2021; Hammer, 1984; Smýkal et al., 2018). PHS refers to the premature germination of seeds within pods that remain attached to the mother plant before harvest (Mares and Mrva, 2014). This phenomenon is closely associated with the absence of fresh seed dormancy (FSD), often assessed through fresh seed germination (FSG) tests, which serve as an indicator of PHS susceptibility (Singh et al., 2012; Lamichaney et al., 2018). PHS is predominantly observed in cereal crops, such as wheat, barley, rice, and sorghum. Nevertheless, it is also a notable phenomenon in certain leguminous crops, including soybean (Dougherty and Boerma, 1984), groundnuts (Nautiyal et al., 2001), black gram (Singh et al., 2012), and mungbean (Ahmad et al., 2014). During the wet harvest season, PHS severely limits crop productivity and quality (Singh et al., 2012; Gupta, 2019). This vulnerability arises from the recirculation of genotypes, primarily for yield in breeding programs, which narrows the genetic base and diminishes dormancy, thereby increasing the risk of PHS. Key factors that influence tolerance to PHS include FSD, hardness of the seed coat, presence of a waxy coating on pods (Mogali et al., 2023), activity of the enzyme α-amylase (Mares and Mrva, 2014), and variations in hormonal responses (Fang and Chu, 2008). These traits are often regulated by specific genes associated with quantitative trait loci that contribute to phenotypic variation (Liu et al., 2013). In Northern and Eastern India, mungbean is primarily cultivated during the rainy season (mid-July to mid-October). Extended periods of rainfall and elevated relative humidity during crop maturation frequently induce premature germination. This phenomenon leads to diminished yield and seed quality, causing significant economic losses to stakeholders (Andrews, 1982; Hampton et al., 2013; Maity et al., 2016; Rashid et al., 2018; Kadam et al., 2022). In extreme cases, yield losses attributed to PHS can reach 60–70% (Durga and Kumar, 1997), causing annual economic losses estimated to be billions of dollars worldwide (Black et al., 2006).

PHS remains a major constraint on yield stability, particularly when rainfall occurs at physiological maturity or immediately before harvest (Rao et al., 2007). Consequently, addressing PHS has become an increasing priority for mungbean producers, breeders, and researchers worldwide (Jia et al., 2024; Pratap et al., 2019; Chauhan et al., 2010). A potential strategy is to introduce a short period of seed dormancy, which can reduce the risk of premature sprouting. Persistent seed dormancy, however, is undesirable because it impedes rapid and uniform seed emergence. Mungbean seeds lack FSD; thus, a brief dormancy period of 10–15 days is considered beneficial for minimizing losses due to PHS.

As the primary wild progenitor of cultivated mungbean, *Vigna radiata* var. *sublobata* represents an essential evolutionary source, contributing unique alleles and genetic diversity that underpin efforts in mungbean improvement programs. Notably, several accessions of *V. radiata* var. *sublobata* have been reported to exhibit tolerance to bruchid beetle infestation (Tomooka et al., 1992; Miyagi et al., 2004; Schafleitner et al., 2016) and resistance to yellow mosaic virus (Pal et al., 2000) and low temperatures (Lawn et al., 1988). This wild species also exhibits beneficial agronomic traits, such as transient hard-seededness and natural resistance to pre-harvest sprouting, which are often lacking in cultivated varieties (Lamichaney et al., 2021; Lawn et al., 1986). Seed dormancy in wild mungbean has been identified as physical, where the seed coat itself prevents water uptake (Laosatit et al., 2022), and in some cases, it is combined, involving both physical and physiological mechanisms that delay germination (Somta et al., 2022). Laosatit et al. (2022) reported that wild mungbean seeds possess a distinct palisade cuticle layer in the seed coat, which is an anatomical feature absent in cultivated varieties and contributes to their enhanced seed dormancy. Humphry et al. (2005) proposed that transferring hard-seededness from wild to cultivated mungbean could significantly mitigate issues related to weather-induced seed damage, particularly in high-humidity environments where PHS is the most problematic. Breeders can develop improved mungbean varieties that are better suited to challenging climatic conditions by understanding and utilizing these seed coat anatomical traits and the inherent dormancy mechanisms. One of the most effective approaches to achieve this transfer is introgression breeding, which involves distant hybridization and selection to incorporate desirable traits of wild relatives into elite cultivars (Thakur et al., 2024). This is an effective method for expanding the genetic diversity in several crops, including mungbean. Notably, introgression breeding has led to the development of several improved mungbean cultivars by broadening the genetic base and enabling the transfer of various agronomically important traits from crop wild relatives into elite backgrounds (Pratap et al., 2021). Introgression breeding demonstrates the pragmatic use of interspecific hybridization for the enhancement of traits of interest in mungbean (Sharma et al., 2022).

Extensive research has been conducted on PHS tolerance in cereals; however, studies on this trait in pulses, particularly in mungbean, are relatively limited. Previous studies have evaluated mungbean germplasm collections to identify genotypes that are tolerant to PHS (Rao et al., 2007; Vijay and Gupta, 2008; Anupama et al., 2012; Ahmad et al., 2014; Lamichaney et al., 2017; Gupta et al., 2024). However, no study has evaluated crosses developed from inter-specific derivatives (lines) with a known wild background in the context of PHS. To the best of our knowledge, this is the first study to develop and evaluate such crosses for PHS tolerance in mungbean. The present study was designed with the following major objectives: (1) to quantify the genetic variation in PHS and FSG tolerance across genotypes and evaluate the association between these traits, (2) to establish a robust classification system for genotype tolerance levels, and (3) to elucidate the genetic architecture underlying seed dormancy. By systematically investigating these component traits, our findings provide a foundational framework for marker-assisted selection and targeted gene pyramiding, thereby accelerating the development of high yielding, climate-resilient mungbean cultivars with enhanced resistance to PHS, a vital advancement for tropical and subtropical agroecosystems that face increasing variability in rainfall.

## 2 Materials and methods

### 2.1 Development of genotypes from pre-breeding program

The study was conducted at the Agriculture Research Station, Badnapur, situated in the Marathwada region of Maharashtra, India (19.86902, 75.70495), which is characterized by a semi-arid climate and an average annual rainfall of approximately 750mm, particularly dominant in June–September.

**Figure 1:**
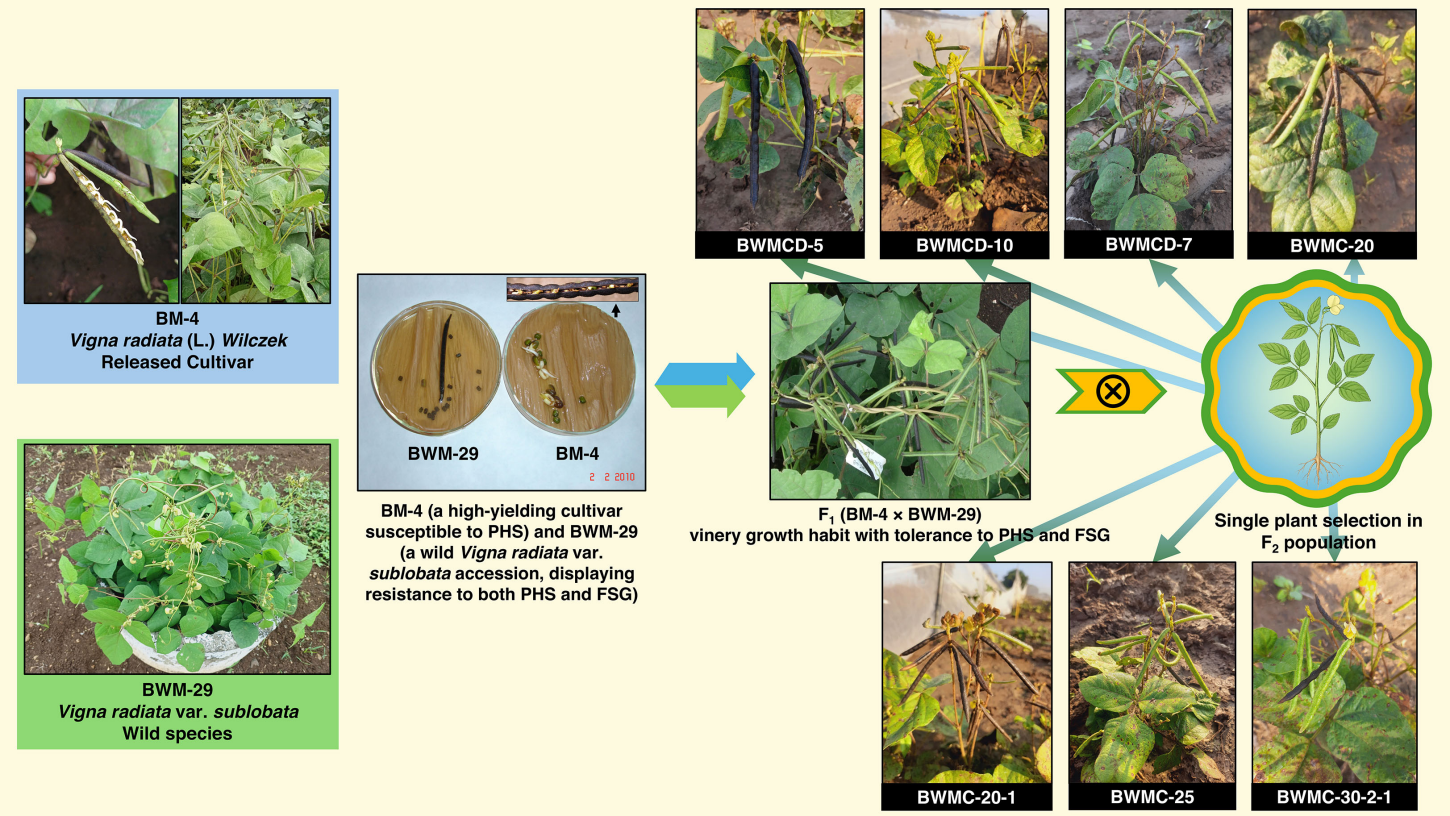
Initiation and advancement of the pre-breeding program in 2010 through an inter-specific cross between BM-4 (a high yielding, PHS susceptible cultivar) and BWM-29 (wild, PHS and FSG resistant accession). Single plant selection was performed in the F_2_ generation, leading to the identification of stable F_10_ mungbean genotypes (wild derivatives) exhibiting combined seed dormancy and high yield traits. These stable genotypes were used as male parents in this study to introgress the target traits into commercially released mungbean varieties, as depicted in **Table 1**.

In the rainy season of 2010, a pre-breeding program was initiated by crossing BM-4 (a high yielding cultivar susceptible to PHS) with BWM-29 (wild *Vigna radiata* var. *sublobata* accession, which displayed resistance to both PHS and FSG). The F_1_ plants exhibited a vinery (climbing or twining type) growth habit and tolerance to PHS and FSG (**Figure 1**). These plants were then advanced to produce the F_2_ population. Single-plant selection was carried out in the F_2_ population during the rainy season of 2012. The progenies from these populations were subsequently evaluated for PHS and other yield-related traits in each generation. During the rainy season of 2020, seven stable and non-segregating genotypes (wild derivatives) were identified from the F_10_ generation. These stable derivatives showed PHS tolerant trait from the wild parent with favorable agronomic characteristics from the cultivated line (**Supplementary Table S1**; **Figure 1**).

### 2.2 Hybridization, development of F_1_ and backcross progenies

**Figure 2:**
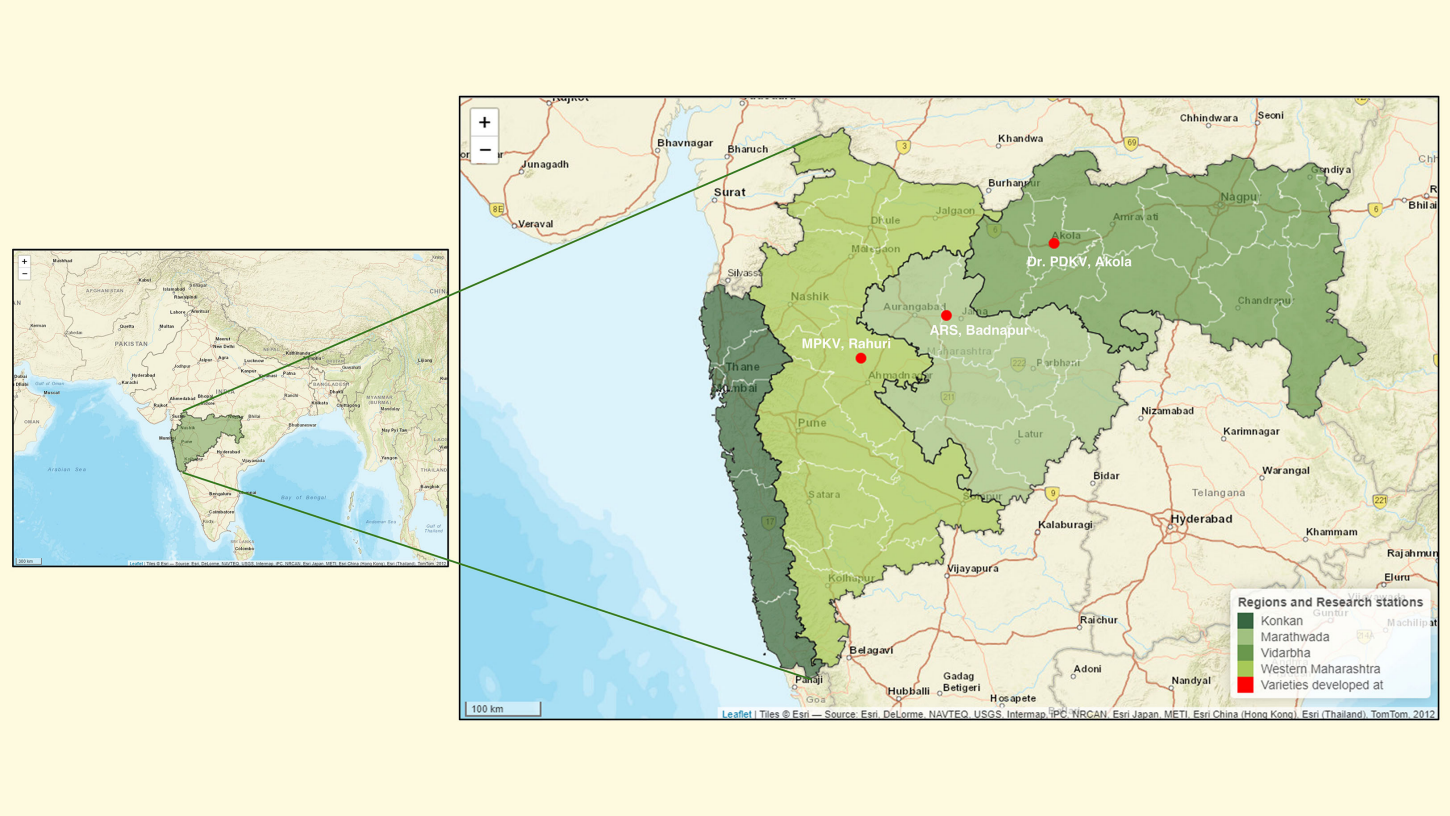
Map of Maharashtra state in India showing major agroclimatic regions categorized by average annual rainfall. The state is divided into four broad zones: Konkan (>2500mm rainfall), Western Maharashtra (750–1200 mm), Vidarbha (700–1600 mm), and Marathwada (500–900 mm). These rainfall zones reflect varying levels of suitability for mungbean cultivation. Red bullet points indicate the locations of agriculture research stations where the cultivars (female parent used in this study) were originally developed and released, including ARS Badnapur (Marathwada), Dr. PDKV Akola (Vidarbha), and MPKV Rahuri (Western Maharashtra). Basemap source: Esri. ‘World Street Map.’ (https://www.arcgis.com/home/item.html?id=de26a3cf4cc9451298ea173c4b324736).

The six female parents used in this experiment were commercially released mungbean cultivars that have been widely adopted in different agroclimatic regions of Maharashtra. These cultivars are known for their high yield potential but are vulnerable to PHS and FSG. The selected cultivars represent diverse regional environments: ARS Badnapur (Marathwada region) with low and erratic rainfall (500–900 mm annually); Dr. PDKV Akola (Vidarbha region), characterized by a semi-arid to moderate rainfall pattern (700–1600 mm); and MPKV Rahuri (Western Maharashtra), which experiences moderate rainfall and lies in a rain-shadow zone (750–1200 mm). Mungbean cultivation is rare in the Konkan region because of the high annual rainfall that exceeds 2500mm (**Figure 2**).

Hybridization was conducted during the rainy season of 2021. Seven stable derivatives obtained from the pre-breeding program were used as male parents and crossed with six distinct, regionally adapted, high yielding cultivars. Thus, a total of forty-two F_1_ hybrids were produced. For hybridization, the anthers of the female parent plants were hand-emasculated the evening before anthesis. The following morning, pollen from the male parent plants was manually dusted onto the stigmas of the emasculated flowers to facilitate controlled cross-pollination. The salient features of the parental lines used in hybridization experiments are listed in **Table 1**.

**Table 1:** Pedigree, origin, and key traits of parental genotypes used in the study.

SN	Parents	Selection/pedigree	Origin	Features
Female parents				
1	BM-4	Selection from the mutation population of T 44	ARS, Badnapur	Short podded, dull green, medium-sized seed, and high yielding
2	BM 2002-1	Selection from the segregating generation of cross JL 781 × BM-4	ARS, Badnapur	Dull and bold seeded, synchronous maturity, and high yielding
3	Phule Chetak	Selection from the segregating generation of cross SML 668 × Naval	MPKV, Rahuri	Shiny, seeded, long podded, and bold-seeded, high yielding
4	Kopergaon	Selection from bulk local germplasm.	Dr. PDKV, Akola	Shiny and bold seeded.
5	AKM 4	Selection from the segregating generation of cross BM-4 × PS16	Dr. PDKV, Akola	Short podded and high yielding
6	PKV Green Gold	Released cultivar	Dr. PDKV, Akola	Shiny seed, tolerant to powdery mildew, and high yielding.
Male parents				
7	BWMCD-5	Derivatives of inter-specific cross between BM-4 × BWM-29 (*Vigna radiata* var. *sublobata*)	ARS, Badnapur	Bold seeded, tolerant to PHS
8	BWMCD-10			Bold seeded, tolerant to PHS
9	BWMCD-7			Bold seeded, tolerant to PHS
10	BWMC-20			Long podded, tolerant to PHS
11	BWMC-20-1			Long podded, tolerant to PHS
12	BWMC-25			Long podded, tolerant to PHS
13	BWMC-30-2-1			Black seeded, long podded, tolerant to PHS

PDKV, Akola, Dr. Panjabrao Deshmukh Krishi Vidyapeeth, Akola; ARS, Badnapur, Agriculture Research Station, Badnapur; MPKV, Rahuri, Mahatma Phule Krishi Vidyapeeth, Rahuri.

To understand the genetic pattern of seed dormancy, two distinct parental expression traits (dormant and non-dormant) were selected for genetic analysis. The F_1_ plants from each cross combination were allowed to self-pollinate naturally to produce the F_2_ generation. Simultaneously, these F_1_ individuals were backcrossed with their respective parents to produce two backcross populations, BC_f_ (F_1_ × female parent) and BC_m_ (F_1_ × male parent).

### 2.3 Phenotyping and laboratory evaluation

In the rainy season of 2022, 55 genotypes (comprising 13 parental lines and 42 cross combinations) were grown in the field using a randomized block design with two replications. Each genotype was planted in two rows per plot, with a row length of 4m. The spacing arrangement was maintained at 45cm between rows, and 10cm between plants within a row. We began phenotyping just before physiological maturity, marked by the first pods turning from green to black. The weather conditions during the 2022 rainy season are summarized in **Supplementary Table S2**. To study the PHS, ten pods from each genotype were placed in Petri dishes lined with water-soaked germination paper. Following the culturing of the pods, the Petri dishes were incubated at 25 °C. The percentage of seed germination within a pod was recorded after five days of incubation.

Seed germination within a pod served as a method to evaluate PHS tolerance, where a higher PHS% indicated a higher susceptibility to PHS. The number of germinated seeds was recorded for each replicate, and the percentage of germinated seeds was calculated as follows:


$$PHS\;\left(\%\right)=\frac{Number\;of\;germinated\;seeds\;in\;pods}{Total\;number\;of\;pods}\times 100$$

Based on this observation, we developed a scale to measure pre-harvest sprouting, which provides a tolerance level according to the PHS%.

For FSG, pods were harvested prior to physiological maturity and manually threshed. Freshly harvested seeds of all genotypes were immediately brought to the laboratory for FSG testing. For each genotype, 50 seeds from two replications were placed in Petri dishes. The seeds were incubated at 25 °C. FSG (%) was recorded after seven days of incubation, according to Rao (2006). Seed germination in a petri dish was used to measure FSG tolerance, where a higher FSG% indicated a higher susceptibility, and vice versa.


$$FSG\;\left(\%\right)=\frac{Number\;of\;fresh\;seeds\;germinated}{Total\;number\;of\;fresh\;seeds\;incubated}\times 100$$

For genetic studies, parents with their two F_1_ progenies, F_2_ progenies, and backcross populations (BC_f_ and BC_m_) were sown during the 2023 rainy season. Fresh seed dormancy was evaluated in all six generations. Seeds that germinated for seven days were classified as non-dormant, whereas those that germinated after seven days were classified as dormant.

### 2.4 Statistical analysis

The mean differences in FSG and PHS among the genotypes were recorded in petri dishes. The data recorded from the laboratory experiments were analyzed according to the procedure suggested by Panse and Sukhatme (1967).

Data preprocessing and trait scaling were performed using R packages. Principal Component Analysis (PCA) was conducted in RStudio (Posit Team, 2025) using the ‘plotly’ package (Sievert, 2020). Correlation analysis was conducted using Pearson’s correlation coefficient method (Pearson, 1920), and the correlation plot was generated with the ‘ggplot2’ package (Wickham, 2016). A two-way clustered heatmap illustrating the relationships between traits and genotype accessions was created using the ‘pheatmap’ package (Kolde, 2018). The map was created using RStudio (Posit Team, 2025) and the ‘leaflet,’ ‘sf,’ and ‘geodata’ packages (Cheng et al., 2025; Pebesma and Bivand, 2023; Hijmans et al., 2025) with ESRI basemap for visualization. To elucidate the genetic basis of seed dormancy, the expected trait values for the F_2_ generation were derived using the predicted Mendelian ratios and compared with the observed values. The differences between these observed and expected outcomes were analyzed using the chi-square (χ2) test to evaluate the fit of the data. This approach allowed us to assess whether the segregation patterns observed in the F_2_, BC_m_, and BC_f_ generations were aligned with the proposed genetic models. By applying the χ2 test, we aimed to verify the consistency of inheritance patterns across all the studied populations. The procedure for the χ2 analysis followed the methodology outlined by Panse and Sukhatme (1967) as follows:


$${\text{χ}}^{2}=\sum \frac{{\left(O-E\right)}^{2}}{E}$$

where O is the observed value and E is the expected value. Deviations were considered significant only when the calculated chi-square value was greater than the tabulated value at (n−1) degree of freedom and 0.05 level of significance (alpha).

## 3 Results

### 3.1 Genetic analysis for PHS and FSG

#### 3.1.1 Experimental variance partitioning and variability studies

Analysis of variance indicated significant differences among all genotypes for the traits studied (**Table 2**). Descriptive statistics for each trait are presented in **Table 3**. A wide range of variations in both PHS and FSG was observed among mungbean genotypes. In all female parent lines, both fresh seeds and those within intact pods absorbed water within 2–3 h and initiated germination within 24 h, indicating their susceptibility to PHS. In contrast, the male parents, which were inter-specific derivatives, displayed delayed germination, with some initiating after up to 13 days, reflecting a greater tolerance. The FSG values varied from 8.50% in the male parent BWMC-30-2–1 to 100% in the female parent BM-4, with a mean of 52.18%. Similarly, the PHS values ranged from 9.45% in BWMC-30-2–1 to 100% in BM-4, with a mean of 51.59% (**Supplementary Table S1**). Among male parents, the lowest FSG and PHS were recorded in BWMC-30-2-1 (8.50% and 9.45%, respectively), followed by BWMC-20-1 (9.45% FSG, 12.88% PHS), and BWMCD-5 (11.00% FSG, 12.13% PHS), indicating higher levels of tolerance. Across the 42 crosses evaluated, FSG values ranged from 4.76% (BM 2002-1 × BWMCD-10) to 95.40% (BM-4 × BWMC-20), whereas PHS ranged from 3.55% (Kopergaon × BWMC-30-2-1) to 98.86% (BM-4 × BWMC-20) (**Supplementary Table S1**).

**Table 2:** Analysis of variance for the morpho-physiological traits studied in mungbean accessions.

SN	Traits	Mean sum of square		
	Source	Replications	Genotypes	Error
	df	1	54	54
1	DF	0.082	9.365***	1.415
2	DM	0.445	16.37***	3.908
3	PH	2.903	65.048***	3.581
4	NB	0.015	9.034***	0.413
5	NOC	0.132	11.843***	0.827
6	NOP	4.623	125.64***	5.821
7	NOS	0.042	3.496***	0.381
8	PL	0.342	7.317***	0.774
9	100 SW	0.055	1.514***	0.071
10	SY	5.872	28.787***	4.134
11	FSG	0.448	2047.04***	19.192
12	PHS	67.659	2104.072***	28.784

DF, days to 50% flowering; DM, days to maturity; PH, plant height; NB, number of primary branches; NOC, number of clusters per plant; NOP, number of pods per plant; NOS, number of seeds per pod; PL, pod length; 100 SW, 100 seed weight; SY, seed yield; FSG, fresh seed germination; PHS, pre-harvest sprouting; *** denotes the significant at 0.1% probability level.

**Table 3:** Descriptive statistics of morphological traits in mungbean.

Traits	Minimum	Maximum	Mean	CV	SE	CD	SD	Skewness	Kurtosis
DF	32.50	40.50	36.609	3.25	0.84	2.39	1.19	0.02	1.94
DM	58.50	71.00	66.118	2.99	1.40	3.96	1.98	-0.56	2.98
PH	48.88	75.40	60.961	3.10	1.34	3.79	1.89	0.33	3.11
NB	3.20	10.80	7.342	8.75	0.45	1.29	0.64	0.03	1.65
NOC	5.33	13.77	9.574	9.50	0.64	1.82	0.91	0.13	1.84
NOP	12.70	39.90	27.078	8.91	1.71	4.84	2.41	-0.35	1.93
NOS	9.94	16.32	14.006	4.41	0.44	1.24	0.62	-0.54	3.23
PL	8.25	15.49	12.633	6.96	0.62	1.76	0.88	-0.49	2.44
100 SW	3.03	5.98	4.711	5.64	0.19	0.53	0.27	-0.26	1.85
SY	7.27	21.63	15.525	13.10	1.44	4.08	2.03	-0.34	2.07
FSG	4.76	100.00	55.011	7.96	3.10	8.78	4.38	-0.09	1.56
PHS	3.55	100.00	50.960	10.53	3.79	10.76	5.37	0.13	1.53

DF, days to 50% flowering; DM, days to maturity; PH, plant height; NB, number of primary branches; NOC, number of clusters per plant; NOP, number of pods per plant; NOS, number of seeds per pod; PL, pod length; 100 SW, 100 seed weight; SY, seed yield; FSG, fresh seed germination; PHS, pre-harvest sprouting; CV, coefficient of variation; SE, standard error; CD, critical difference; SD, standard deviation.

Based on the mean percentage of seed germination inside pods, which was used to measure PHS tolerance, of the 42 crosses, Kopergaon × BWMC-30-2-1 (3.55%), Kopergaon × BWMC-20-1 (5.15%), BM 2002-1 × BWMCD-10 (6.50%), and BM-4 × BWMCD-5 (8.30%) exhibited tolerance (<10%), indicating minimal pod damage from PHS (**Supplementary Table S1**). These crosses also stood out by recording less than 10% FSG even after 13 days of incubation. Additionally, these crosses exhibited a hard-seeded phenotype, as observed in seed coats. The tolerance of PHS in these crosses may be attributed to the presence of hard-seed coats.

#### 3.1.2 Correlation coefficient analysis

**Figure 3:**
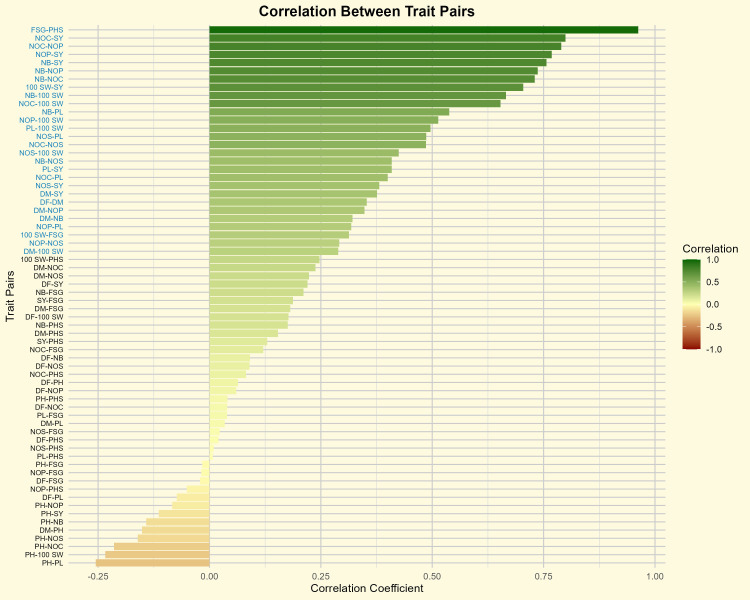
Correlation coefficients between trait pairs in mungbean. Trait pairs shown in blue color font indicate statistically significant correlations (P< 0.05). The color gradient represents the strength and direction of correlation, from negative (red) to positive (green). DF, days to 50% flowering; DM, days to maturity; PH, plant height; NB, number of primary branches; NOC, number of clusters per plant; NOP, number of pods per plant; NOS, number of seeds per pod; 100 SW, 100 seed weight; PL, pod length; SY, seed yield; FSG, fresh seed germination; PHS, pre-harvest sprouting.

The results showed a strong and significant positive correlation between the PHS and FSG, with a coefficient of 0.96 (**Figure 3**; **Supplementary Table S3**). Despite the significant positive correlation observed between the in two traits, several crosses, including BM-4 × BWMC-30-2-1 (15.00%), BM 2002-1 × BWMC-30-2-1 (17.15%), AKM 4 × BWMC-20-1 (18.00%), AKM 4 × BWMCD-5 (23.00%), Phule Chetak × BWMC-20 (26.33%), PKV Green Gold × BWMC-30-2-1 (22.06%), and Kopergaon × BWMCD-7 (28.50%) recorded PHS levels between 15-30%. However, these crosses exhibited relatively higher FSG percentages when seeds were manually extracted from the pods. This suggests that pod morphological traits, such as epicuticular wax layer and pod wall thickness, may act as physical barriers to moisture uptake, thereby limiting PHS occurrence. These findings support the hypothesis that pod-related traits play a substantial role in conferring tolerance to PHS independent of intrinsic seed dormancy.

**Figure 4:**
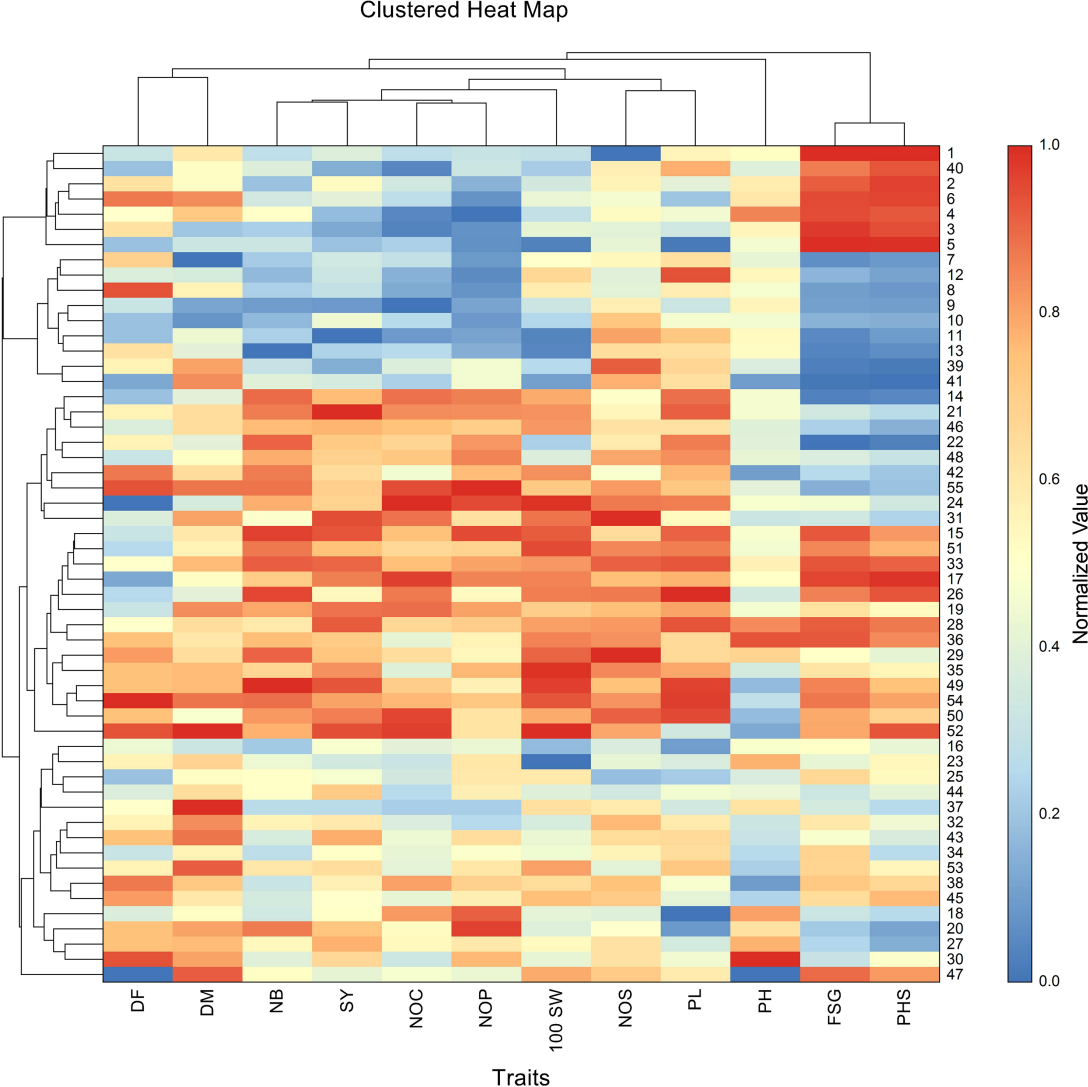
Clustered heatmap shows normalized trait values across 55 mungbean genotypes. For visualization, the values for each trait were independently scaled to a range of 0 (minimum value) to 1 (maximum value), where high values are shown in red and low values in blue. Both genotypes and traits were clustered using Euclidean distance and the Group Average (Unweighted Pair-Group) clustering method. Notably, PHS and FSG clustered together, highlighting their co-expression across genotypes. The heatmap also reveals distinct genotype groupings with similar multi-trait profiles. The numbers shown in the figure represent genotype codes, corresponding to genotype numbers listed in **Supplementary Table S1**. Traits are mentioned as, DF, days to 50% flowering; DM, days to maturity; PH, plant height; NB, number of primary branches; NOC, number of clusters per plant; NOP, number of pods per plant; NOS, number of seeds per pod; 100 SW, 100 seed weight; PL, pod length; SY, seed yield; FSG, fresh seed germination; PHS, pre-harvest sprouting.

The highest pod loss due to PHS was observed in the crosses, including BM-4 × BWMC-20 (98.86%), followed by BM 2002-1 × BWMC-25 (93.54%), PKV Green Gold × BWMC-20 (93.50%), Kopergaon × BWMC-25 (93.27%), Phule Chetak × BWMC-25 (91.27%), Phule Chetak × BWMCD-5 (87.83), Kopergaon × BWMCD-10 (84.78%), and BM-4 × BWMCD-10 (82.41%) (**Table 2**). Notably, PHS-tolerant genotypes were strategically used as male parents in hybridization; however, only a few of the resulting F_1_ crosses showed effective tolerance to PHS. Furthermore, the 100 seed weight was positively correlated with FSG (r = 0.31) and PHS (r = 0.25). These results suggest that genotypes with larger/bold seeds have a slight tendency toward germination and a higher risk of PHS development. This trend was further supported by the clustered heatmap (**Figure 4**), which revealed that PHS and FSG clustered together, indicating similar expression profiles across the genotypes.

Cluster analysis of the normalized trait data across genotypes revealed distinct patterns of trait association and genotype grouping. The dendrogram, depicting hierarchical clustering, organized the genotypes into several clusters based on similarity in trait expression, with clear differences observed between high- and low-performing groups (**Figure 4**). Notably, traits such as days to flowering, number of primary branches, number of clusters per plant, number of pods per plant, number of seeds per pod, pod length, 100 seed weight, and seed yield clustered together, indicating their co-variation and potential interdependence. Conversely, PHS and FSG formed a separate cluster, showing inverse patterns relative to yield-related traits. Several genotypes exhibited concurrently high values for PHS and FSG, but lower values for the number of pods, days to flowering, and number of pods, suggesting a potential trade-off between sprouting susceptibility and agronomic performance.

#### 3.1.3 Principal component analysis

**Figure 5:**
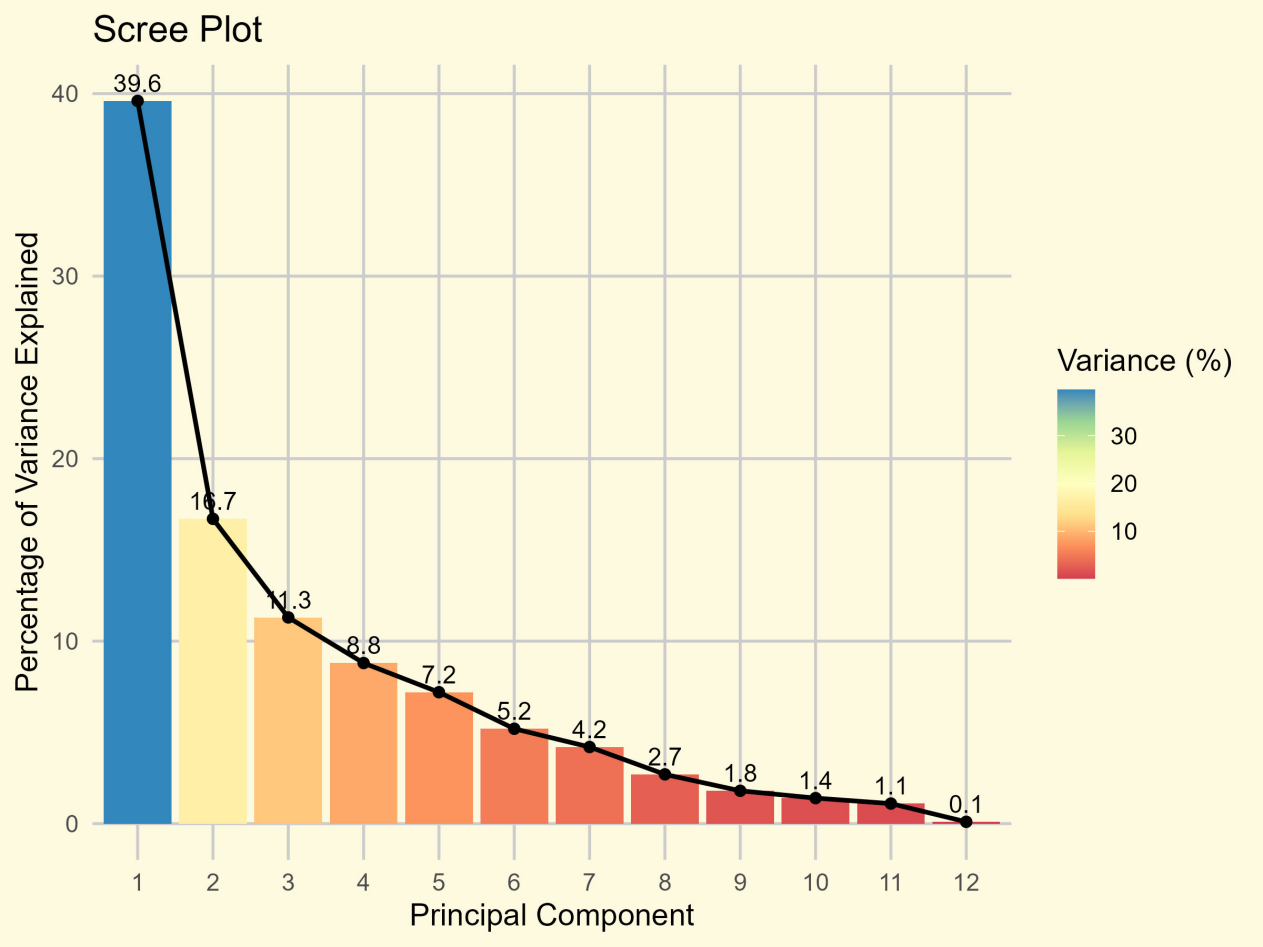
Scree plot of 10 principal components (PCs) against their eigenvalues.

Of the 12 traits utilized in principal component analysis, only the first four principal components (PCs) with eigenvalues over 1 accounted for most of the variability, representing approximately 76.18% of the variability among the traits in the examined accessions (**Figure 5**; **Supplementary File S4**). Therefore, these four PCs accorded significant emphasis for further elucidation in this study.

PCA of the traits revealed that seed yield had the highest loading on the first principal component, with a value of 0.409, indicating its strong contribution to the variation captured by this component (**Supplementary File S5**). In the second principal component, the PHS exhibited the highest loading of 0.674, highlighting its dominant role in driving variation along that axis. Additionally, days to 50% flowering showed the highest loading in PC3 with 0.668, whereas plant height was the main contributor to both PC4 and PC5, with loadings of 0.683 and 0.567, respectively. These findings demonstrate that different traits distinctly contribute to each principal component, with PHS explaining the greatest variability in PC2, highlighting its importance in the structure of multivariate trait variation.

**Figure 6:**
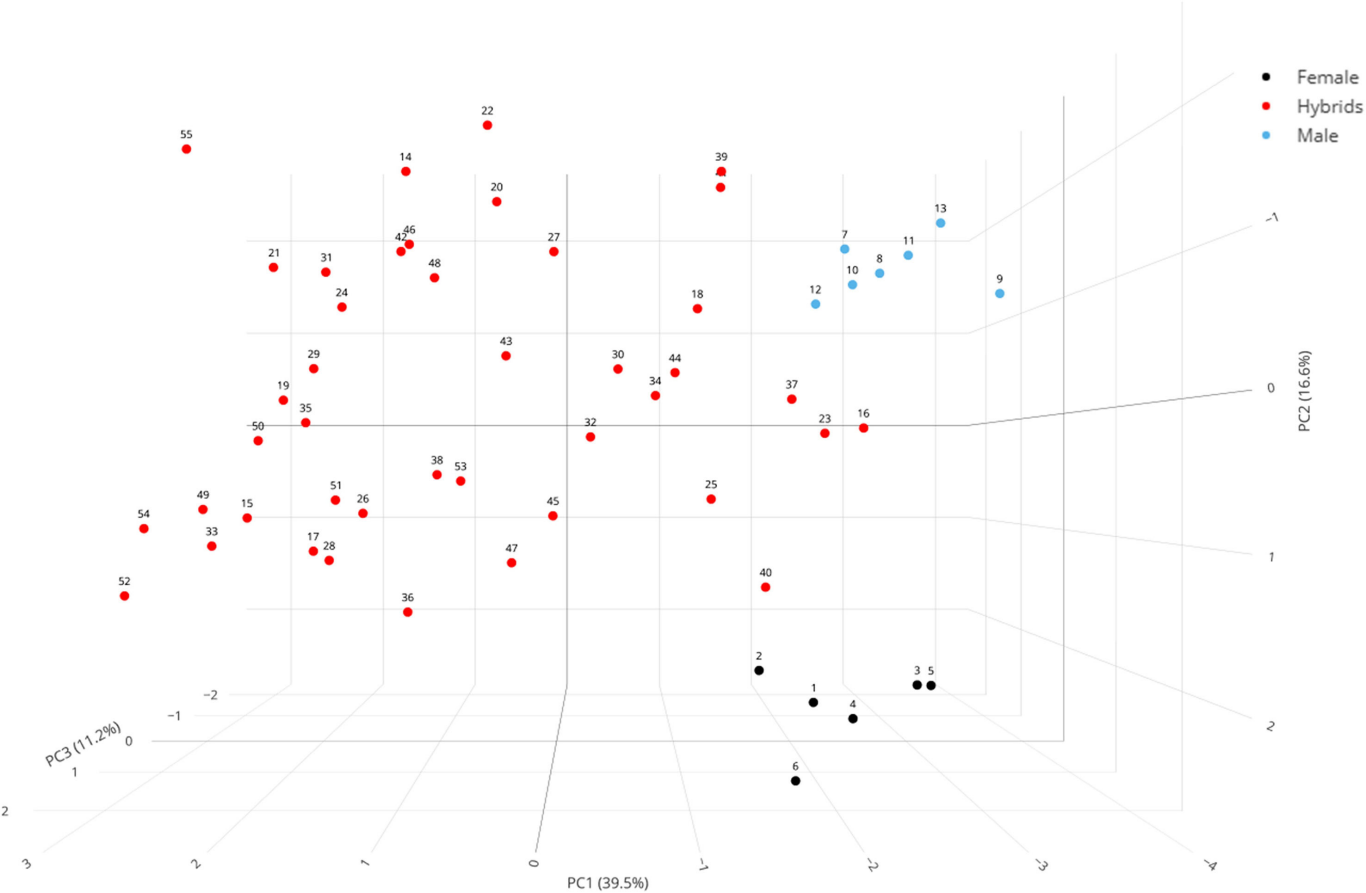
Principal Component Analysis (PCA) of 55 mungbean genotypes based on 12 agro-morphological traits. The 3D plot shows the genetic divergence between female parents (black dots, n = 6), male parents (blue dots, n = 7), and their F_1_ hybrids (red dots, n = 42). The first three components explain 67.4% of the total phenotypic variance. PC1 (39.5%) is primarily driven by seed yield, separating genotypes based on productivity. PC2 (16.6%) is mainly associated with PHS, and PC3 (11.2%) is most influenced by days to 50% flowering. The clear separation of the parental clusters from the widely dispersed hybrids highlights the generation of novel phenotypic variation. Genotype numbers correspond to those in **Supplementary Table S1**.

To visualize the distribution of genotypes, a 3D PCA plot was generated using the first three principal components (**Figure 6**). This plot illustrates that the genotypes were widely dispersed in a three-dimensional space, particularly along PC1 (driven by seed yield), PC2 (driven by PHS), and PC3 (driven by days to 50% flowering), demonstrates that the crosses successfully generated significant phenotypic variation and novel combinations of these key agro-morphological traits. The genotypes located closer to each other in the plot exhibited less variability, whereas those farther apart were more divergent. Interestingly, genotypes positioned near each other also appeared to form distinct clusters, further confirming the genotype groupings observed in the clustered heatmap (**Figure 4**), thereby reinforcing the consistency of the multivariate trait patterns across both approaches.

### 3.2 Categorization and breeding strategy for pre-harvest sprouting tolerance

The 42 crosses were classified into four categories based on PHS tolerance, providing a practical framework for selecting breeding lines with improved tolerance. The categories were defined as follows: tolerant (<10% sprouting), moderately tolerant (10–30%), moderately susceptible (30–60%), and susceptible (>60%) (**Table 4**). Of the 42 crosses, four were classified as tolerant, 11 as moderately tolerant, 12 as moderately susceptible, and 15 as susceptible.

**Table 4:** Categorization of mungbean crosses based on tolerance to pre-harvest sprouting.

PHS category	PHS (%)	Crosses
Tolerant	<10	BM-4 × BWMCD-5, BM 2002-1 × BWMCD-10, Kopergaon × BWMC-20–1 and Kopergaon × BWMC-30-2-1
Moderately tolerant	10-30	BM-4 × BWMC-20-1, BM-4 × BWMC-25, BM-4 × BWMC-30-2-1, BM 2002-1 × BWMCD-5, BM 2002-1 × BWMC-30-2-1, Phule Chetak × BWMC-20, Phule Chetak × BWMC-30-2-1, Kopergaon × BWMCD-7, AKM 4 × BWMCD-5, AKM 4 × BWMC-20-1, PKV Green gold × BWMC-30-2-1
Moderately susceptible	30-60	BM-4 × BWMCD-7, BM 2002-1 × BWMCD-7, BM 2002-1 × BWMC-20, BM 2002-1 × BWMC-20-1, Phule Chetak × BWMCD-10, Phule Chetak × BWMCD-7, Phule Chetak × BWMC-20-1, Kopergaon × BWMCD-5, AKM 4 × BWMCD-10, AKM 4 × BWMCD-7, AKM 4 × BWMC-30-2–1 and PKV Green gold × BWMC-20-1
Susceptible	>60	BM-4 × BWMCD-10, BM-4 × BWMC-20, BM 2002-1 × BWMC-25, Phule Chetak × BWMCD-5, Phule Chetak × BWMC-25, Kopergaon × BWMC-10, Kopergaon × BWMC-20, Kopergaon × BWMC-25, AKM 4 × BWMC-20, AKM 4 × BWMC-25, PKV Green gold × BWMCD-5, PKV Green gold × BWMCD-10, PKV Green gold × BWMCD-7, PKV Green gold × BWMC-20 and PKV Green gold × BWMC-25

Cross combinations in the tolerant category can be utilized in mungbean improvement programs to produce elite cultivars with enhanced PHS tolerance. Moderately tolerant crosses can contribute valuable genetic diversity for refining traits, such as seed dormancy, helping to broaden the genetic base. Moderately susceptible crosses may possess partial resistance and can be utilized in pre-breeding programs to dissect complex dormancy-related traits and pod-associated mechanisms, serving as a bridging material for improving PHS tolerance through further selection and stabilization. Susceptible crosses were excluded to avoid perpetuation of undesirable traits. This systematic approach enables breeders to make informed decisions about which crosses should be incorporated into their breeding efforts.

### 3.3 Genetics of seed dormancy

**Figure 7:**
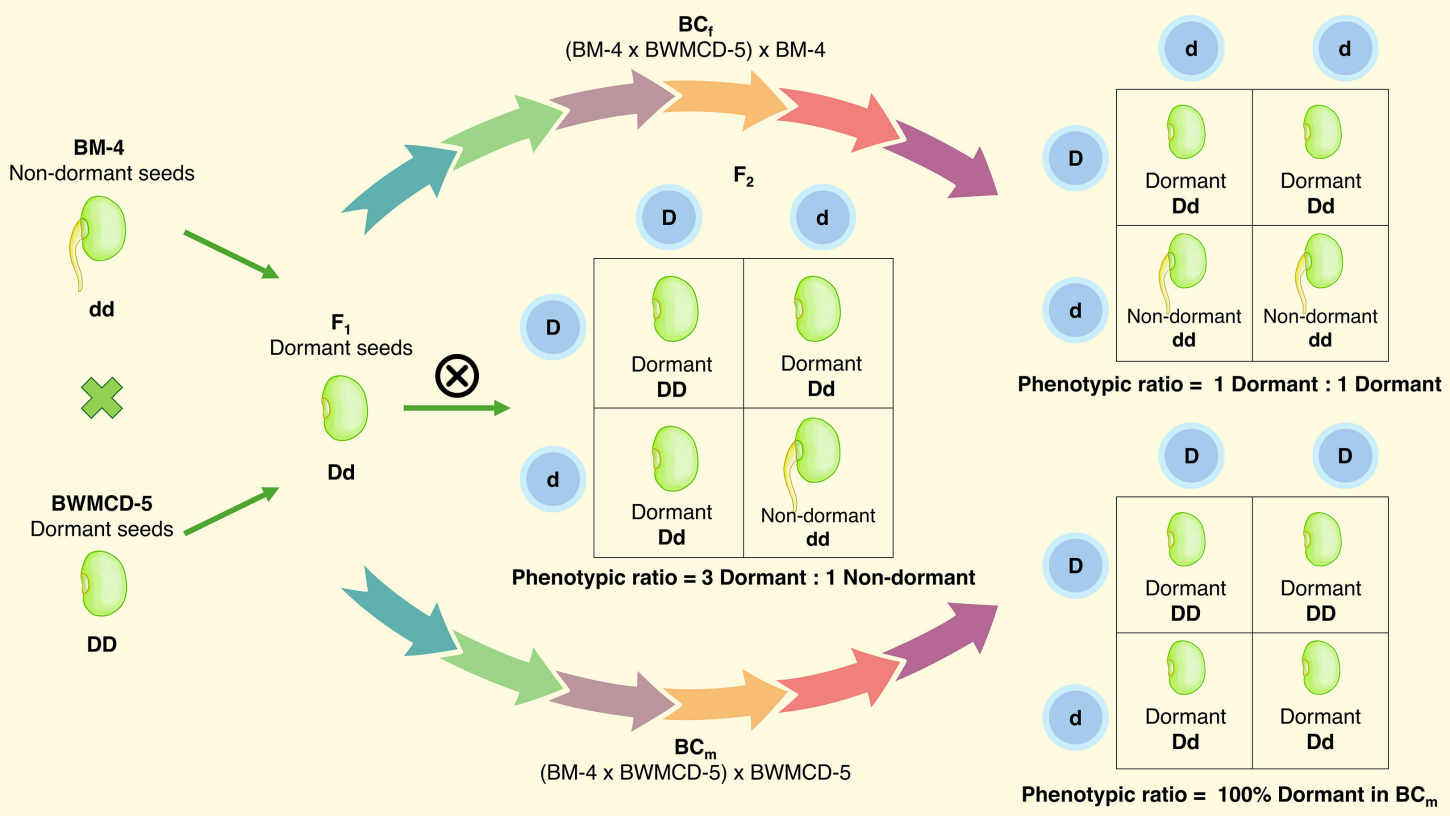
Monogenic ratio for the inheritance of fresh seed germination in mungbean.

The inheritance pattern of seed dormancy has been studied in crosses between dormant and non-dormant parents. Female parents BM-4 and BM 2002–1 were non-dormant, whereas the male parents BWMCD-5 and BWMCD-10 were dormant (**Figure 7**). Crosses between dormant and this parental trait combination (BM-4 × BWMCD-5 and BM 2002-1 × BWMCD-10) produced dormant seeds in the F_1_ generation, in terms of the number of days to germination. The resulting F_1_ generations from these crosses exhibited dormant characteristics, as evidenced by their failure to germinate within seven days, indicating that dormancy is a dominant trait over non-dormancy. The results of the two crosses analyzed for fresh seed dormancy inheritance are shown in **Table 5**. In the F_2_ generation of the BM-4 × BWMCD-5 cross, 86 seeds were dormant, and 22 were non-dormant. This segregation closely fits the expected 3:1 ratio for dormant to non-dormant (χ² = 1.234, P=0.266). The observed segregation in the F_2_ generation was further confirmed by analyzing backcross generations. In the BC_m_ generation, all 18 seeds were dormant, whereas in the BC_f_ generation, 14 seeds were dormant and 10 were non-dormant, fitting the expected 1:1 ratio (χ² = 0.667, P=0.414). Similarly, in the BM 2002-1 × BWMCD-10, of the 130 F_2_ seeds, 104 were dormant and 26 were non-dormant. This segregation also fits the expected 3:1 ratio (χ² = 1.733, P=0.187). The BC_m_ generation of this cross showed all 20 dormant seeds, whereas in the BC_f_ generation, 15 of 30 seeds were dormant and 13 were non-dormant. This result also demonstrated a good fit with the expected 1:1 ratio of dormant to non-dormant seeds (χ² = 0.142, P = 0.705). In both crosses, segregation in the F_2_ generation followed a 3:1 ratio of dormant to non-dormant seeds. The backcross progenies with non-dormant parents fit the expected 1:1 ratio. In contrast, all progeny from the backcrosses with dormant parents were dormant. The observed 3:1 segregation in the F_2_ and 1:1 ratio in the BC_f_ generation indicated that fresh seed dormancy in the BM-4 × BWMCD-5 and BM 2002-1 × BWMCD-10 crosses was under monogenic control, with dormancy being dominant over non-dormancy.

**Table 5:** Segregation for seed dormancy (dormant or non-dormant) in F_2_ and backcross populations.

Cross and generation	Total seeds	Observed		Expected		Ratio tested	χ^2^ value	*P*
		Dormant	Non- dormant	Dormant	Non- dormant			
BM-4 (P1)	20	0	20					
BWMCD-5 (P2)	22	22	0					
F_1_	12	12	0					
F_2_	108	86	22	81	27	3:01	1.234	0.266
BC_f_ (F_1_ × BM-4)	24	14	10	12	12	1:01	0.667	0.414
BC_m_ (F_1_ × BWMCD-5)	18	18	0	18	0	1:00	0.000	1.00
	Combined fit of segregation data to model						1.901	0.754
BM 2002-1 (P1)	20	0	20					
BWMCD-10 (P2)	16	16	0					
F_1_	14	14	0					
F_2_	130	104	26	97.5	32.5	3:01	1.733	0.187
BC_f_ (F_1_ × BM 2002-1)	30	15	13	14	14	1:01	0.142	0.705
BC_m_ (F_1_ × BWMCD-10)	20	20	0	20	0	1:00	0.000	1.00
	Combined fit of segregation data to model						1.875	0.760

## 4 Discussion

Breeding for pre-harvest sprouting tolerance poses considerable challenges owing to its polygenic nature and the significant impact of genotype-by-environment interactions (Li et al., 2021). PHS is primarily influenced by environmental conditions and modulated by a range of factors, including morphological traits (King and Richards, 1984; Jaiswal et al., 2012; Lin et al., 2016; Rodriguez et al., 2021), as well as biochemical, hormonal, physiological, and genetic mechanisms (Tai et al., 2021; Vetch et al., 2019). Identifying genotypes that not only exhibit tolerance to PHS but also maintain high yield potential is essential for reducing pre-harvest losses and enhancing seed quality. While extensive research has been conducted on PHS tolerance in cereal crops, there remains a notable gap in the literature regarding pulses, particularly in mungbean. Therefore, this study aimed to assess the genetic variability of PHS tolerance among a diverse set of mungbean genotypes and explore the relationships between PHS and agronomic traits. The mean performance of the parental lines and crosses indicated a wide range of variations that could be utilized by including them in mungbean breeding programs. The observed wide variability in PHS and FSG closely mirrors the findings of Singh et al. (2017), who studied 20 diverse mungbean genotypes and reported a range of 2.07% to 99.9% for PHS. Lamichaney et al. (2021) screened 59 *Vigna* accessions for PHS and FSG, and reported ranges from 0% to 100%, with means of 55.86% and 61.19%, respectively. Rao et al. (2023) identified that the genotypes LGG 450 and K 851 had lower moisture absorption scores, which resulted in fewer sprouted seeds per pod and indicated greater tolerance to the PHS. Reddy et al. (2025) studied 60 genotypes and noted the germination % ranged from as low as 6.0% to as high as 95.0%, with a pooled mean of 75.63%, indicating significant genotypic diversity in tolerance to PHS.

Six inter-specific wild derivatives (lines) exhibited PHS incidence below 20%, with BWMC-30-2–1 showing the lowest level, with less than 10% PHS. Previous studies have also reported that wild accessions exhibit a higher tolerance to PHS (Ahmad et al., 2014; Sunayana et al., 2013). Similarly, high genetic variation in PHS has been reported in wild *Vigna* sp (Lamichaney et al., 2021), urdbean (Lamichaney et al., 2023), soybean (Kumar et al., 2021), and mungbean (Lamichaney et al., 2018). Modern mungbean accessions have undergone extensive human and natural selection, which has inadvertently favored traits such as higher seed yield while reducing fewer desirable characteristics. Consequently, traits associated with physical seed dormancy, such as hard seed coat, hard pod shell, epicuticular wax accumulation, and genetically controlled dormancy characteristics, were diminished in many genotypes (Laosatit et al., 2022). Although this has resulted in higher yields, it has also made current cultivars more prone to various abiotic stresses, including a greater susceptibility to PHS. The observed variation in PHS among the crosses was moderate to high, with values ranging from 30% to 100%, and a mean of 51.69%. This indicates that tolerance to PHS is governed by complex inheritance. Moreover, significant genotype × environment interactions for PHS have been reported in crops, such as wheat (Li et al., 2021).

In this study, based on the mean percentage of seed germination inside pods, which was used as an indicator of PHS tolerance, the results highlighted four crosses, namely, Kopergaon × BWMC-30-2-1, Kopergaon × BWMC-20-1, BM 2002-1 × BWMCD-10, and BM-4 × BWMCD-5, were recorded less than 10% PHS. Additionally, these crosses exhibited a hard-seeded phenotype, as observed in seed coats. Tolerance to PHS in these crosses may be attributed to the presence of hard-seed coats, which are likely to restrict water uptake and reduce the risk of premature germination. This relationship between hard-seededness and PHS tolerance is consistent with previous findings, where mungbean genotypes with a higher proportion of hard seeds were shown to be more tolerant to PHS (Ambhore et al., 2024; Mogali et al., 2023). Harnessing the genetic potential of these crosses provides valuable opportunities for breeding programs. Integrating hard-seededness into new cultivars may pave the way for the development of mungbean varieties that are not only resilient to PHS but also align with consumer preferences. Therefore, these crosses are crucial for minimizing yield losses due to PHS and represent valuable resources for future breeding programs focused on enhancing crop resilience and productivity.

PHS tolerance is a quantitative trait that is influenced by the environment and controlled by many genes (Bailey et al., 1999). In the present study, the strong positive correlation observed between FSG and PHS (r = 0.96) highlights a clear and consistent relationship between these two traits in mungbean. This finding suggests that genotypes with higher FSG are more prone to PHS, likely because of the shared genetic and physiological mechanisms influencing both traits. The clustered heatmap validated this pattern, demonstrating that PHS and FSG had comparable expression patterns across the genotypes. The heatmap validated the correlation between PHS, FSG, and 100 seed weight, while also illustrating unique genotype clusters based on their multi-trait expression profiles. Multiple genotypes demonstrated persistently high values for various traits, although the other genotypes displayed contrasting trends. In contrast to the correlation matrix, which illustrates purely pairwise associations, the heatmap provides a more thorough representation of the distribution and intensity of the traits across all genotypes. Ahmad et al. (2014) and Singh et al. (2017) also reported positive correlations between the FSG and PHS, supporting the robustness of our observations. Similarly, Gupta et al. (2024) found a strong positive correlation (r = 0.79) between these traits, further reinforcing the idea that higher FSG is associated with increased susceptibility to PHS. Lamichaney et al. (2017) also reported a moderate correlation coefficient of 0.46, suggesting that this association was robust and consistent across different genetic backgrounds and environments. This trait–trait and genotype–trait clustering highlights key associations that can inform trait selection and genotype advancement in breeding programs. These findings suggest that FSG could serve as a practical and reliable indicator of PHS susceptibility in mungbean breeding programs. By targeting genotypes with lower FSG, breeders may be able to select for improved PHS tolerance more effectively. This approach can streamline the selection process and enhance the development of resilient mungbean varieties, ultimately contributing to the improved crop stability and productivity under variable environmental conditions.

The application of principal component analysis effectively highlighted the interconnections among the traits and the most significant contributors to overall phenotypic variation (Nalajala et al., 2023; Palaniyappan et al., 2024). Consistent with previous studies, PCA diminished data dimensionality with minimal information loss, indicating that a limited number of PCs represented most of the total variation (Nalajala et al., 2023; Singh et al., 2020). Seed yield, PHS, and days to 50% flowering have been identified as essential factors influencing phenotypic differentiation, providing explicit targets for selection in future breeding programs. Moreover, clustering and multivariate visualization techniques, such as PCA and hierarchical clustering, consistently categorize genotypes exhibiting analogous tolerance traits, thereby validating the efficacy of these methods in evaluating genetic relationships and organizing core collections for prospective breeding (Odong et al., 2013; Sathuri et al., 2023). The alignment of these techniques enhances confidence in the reliability of genotype classification and supports strategic choices for parental selection.

The observed results point to an intriguing dynamic between pod morphology and PHS tolerance in the mungbean. Several crosses, despite exhibiting relatively high FSG when seeds were manually extracted and soaked in water, maintained moderate PHS levels (15–30%) while still enclosed within the pod. Gupta et al. (2024) found that even PHS tolerant genotypes can exhibits FSG, highlighting the key role of pod traits in seed dormancy in resistance. Pramod et al. (2024) and Verma et al. (2024) highlighted the role of water imbibition in establishing favorable conditions for germination before pod dehiscence. This pattern suggests that the physical characteristics of the pod, such as epicuticular wax layers or a thick pod shell, may act as effective barriers, limit moisture entry, and reduce the likelihood of premature germination. Studies have shown that higher pod wax and thick pod wall content reduce water absorption and improve PHS tolerance (William, 1984; Cheralu et al., 1999; Rao et al., 2007; Vijay and Gupta, 2008; Lamichaney et al., 2018). By selecting pod traits, such as higher wax deposition and wall thickness, breeders can develop mungbean varieties that are better equipped to withstand unfavorable weather conditions, thereby reducing yield losses and enhancing crop stability.

Seed weight is often inversely associated with pre-harvest sprouting. Imrie (1987) reported that smaller mungbean seeds exhibited a higher proportion of hard seeds than larger seeds. However, in the present study, 100 seed weight appeared to be positively correlated with both FSG (r = 0.31) and PHS (r = 0.25), suggesting that genotypes with larger seeds tend to have slightly higher germination rates and a higher likelihood of PHS. This pattern is consistent with the observations of Gupta et al. (2024), who also reported that seed size influences PHS behavior in mungbean. Similar relationships have been documented in other legumes; for example, Singh et al. (1991) found that larger blackgram seeds were associated with fewer dormant and hard seeds, while Khare et al. (1995) reported similar findings in soybean. The development of medium to large-seeded mungbean genotypes (100-seed weight >3.5g) with hard-seededness has become a crucial objective in mungbean breeding. However, the genetic linkage between seed size and hard-seededness may explain the difficulty in maintaining stable hard-seededness in large-seeded varieties.

In the present study, although we strategically used PHS-tolerant genotypes as male parents in the hybridization program, several crosses exhibited significant pod loss (>60%) due to PHS. This highlights the intricate nature of gene interactions and the challenges in achieving stable PHS tolerance through conventional breeding. The significant pod loss observed in these crosses suggests that the PHS tolerance is governed by a complex polygenic system, potentially involving non-additive genetic effects, epistasis, and cytoplasmic or maternal influences. Imrie (1987) reported that dormancy is regulated by gene interactions that can be dominant, partially dominant, or recessive depending on the genetic background of the parent. Therefore, the interaction of recessive or partially dominant alleles failed to confer sufficient dormancy, resulting in increased PHS susceptibility in moderate and susceptible categories. The genetic, biochemical, and molecular mechanisms underlying PHS are intricately linked to environmental variables such as temperature and rainfall (Mares and Mrva, 2014; Tai et al., 2021). The heritability of PHS tolerance has been reported to be low in both soybean (Kueneman and Dassou, 1982) and mungbean (Imrie, 1983), underscoring the substantial influence of environmental factors on this trait. Another possible explanation for increased pod losses is the role of α-amylase activity. Elevated α-amylase levels facilitate mobilization of stored starch reserves, thereby providing energy for seed germination. However, excessive α-amylase levels can lead to premature germination and reduced seed viability. In support of this, Lamichaney et al. (2018) reported that susceptible mungbean genotypes exhibited significantly elevated α-amylase activity at both 48- and 72-hours post-germination in comparison to tolerant genotypes. This observation is supported by studies by Krishnasamy and Seshu (1990); Sunayana et al. (2013), and Simsek et al. (2014), all of which found that genotypes with higher seed germination percentage and greater vulnerability to PHS also exhibited elevated α-amylase activity. These consistent findings indicate that elevated α-amylase activity is not simply an effect of PHS but is a major biochemical factor associated with increased susceptibility. Thus, it ultimately points out that seeds with higher α-amylase production are more likely to germinate prematurely, negatively impacting seed quality and overall crop yield.

The classification of mungbean crosses and wild-derived parents based on PHS tolerance provides a systematic framework for breeders to prioritize materials with the highest potential for improving resistance. Identifying tolerant and moderately tolerant crosses immediately expands the toolbox to enhance resilience and yield stability in regions prone to unpredictable rainfall. This targeted integration supports both the development of elite high yielding cultivars and strategic maintenance of genetic diversity for long-term improvement. Additionally, incorporating moderately susceptible crosses into pre-breeding programs offers valuable opportunities for investigating the mechanisms underlying seed dormancy and PHS tolerance, which can inform future breeding strategies. The widespread adoption of PHS-tolerant genotypes facilitates better crop management, reduces post-harvest losses, and improves marketability and storage durability, ultimately benefiting smallholder farmers and bolstering food security. By leveraging this classification system, breeders can efficiently accelerate the development of climate-resilient mungbean varieties and address both the immediate and future challenges facing sustainable production. Understanding the genetic basis of seed dormancy can facilitate the utilization of wild genetic resources for crop improvement (Imrie, 1987; Lawn et al., 1988), which is particularly important in the context of climate change. The present study provides compelling evidence that fresh seed dormancy in mungbean is governed by a simple monogenic inheritance pattern, with dormancy being dominant over non-dormancy. In both crosses analyzed, the F_1_ generation consistently exhibited dormancy, as indicated by delayed germination, regardless of the non-dormant status of the female parents. The segregation ratios observed in the F_2_, and backcross generations closely fitted the expected ratios of 3:1 and 1:1, respectively, further supporting the single-gene control for this trait. Segregation analysis based on Mendelian genetics revealed seed dormancy in the wild mungbean accession, ‘Pantnagar’ (*V. radiata* var. *sublobata*) from India, which contains approximately 95% dormant seeds, is controlled by a single dominant gene, *Hd1* (Singh et al., 1983). Similarly, Lawn et al. (1988) reported that the wild mungbean accession ‘ACC41’ from Australia, with over 95% dormant seeds, is regulated by a single major gene. Recent investigations further support the classical view of dominant, monogenic control of seed dormancy in wild mungbean. For instance, Isemura et al. (2012) identified the seed dormancy locus *Sdwa5.1.1*, which was shown to exert a major effect on dormancy. More recently, Laosatit et al. (2022) fine-mapped the same major QTL, *Sdwa5.1.1+*, and pinpointed the candidate gene *VrKNAT7-1*, a transcription factor associated with physical dormancy in wild mungbean. Their work demonstrated higher expression of *VrKNAT7–1* in dormant-seeded wild accessions compared to non-dormant cultivated varieties, providing new molecular evidence that reinforces the historical single-gene dominance model. The consistency of these results across diverse genetic backgrounds underscores the pivotal role of single-gene inheritance in the regulation of seed dormancy in mungbean. The confirmation of dominant monogenic control has important implications for mungbean breeding programs. This suggests that introgression or the elimination of dormancy traits can be efficiently managed using classical selection strategies. Understanding the genetic basis of dormancy will provide a foundation for future molecular studies aimed at identifying and manipulating the underlying gene(s) responsible for this trait.

## 5 Conclusion

This study highlights the pressing necessity to tackle pre-harvest sprouting in mungbean cultivation, particularly given the rising climate uncertainty. Future losses sustained by PHS are anticipated to increase owing to the heightened variability in weather, especially rainfall, during crop maturation. This study highlights the urgent need to address PHS in mungbean cultivation, particularly in the context of climate change. The identification and incorporation of PHS-tolerant traits from wild mungbean progenitors into high yielding cultivars through targeted pre-breeding strategies offer a promising solution to this problem. The evaluation of crosses derived from the wild *Vigna radiata* var. *sublobata* introgression lines revealed a considerable level of diversity in seed germination, which could be harnessed in mungbean improvement programs. Four cross combinations exhibited high PHS tolerance, representing key targets for mungbean cultivar development. PHS-tolerant male parents BWMC-30-2-1, BWMC-20-1, and BWMCD-5 are recommended for direct utilization in future breeding programs to enhance PHS resistance in elite backgrounds. The significant correlation between FSG and PHS reinforces the physiological link between dormancy to sprouting susceptibility. Furthermore, the monogenic inheritance of dormancy observed in the two cross combinations facilitates the application of marker-assisted selection for efficient selection and pyramiding of PHS resistance genes in the future. The incorporation of PHS-tolerant characteristics using molecular breeding techniques, specifically by identifying genes linked to fresh seed dormancy from wild donors and introgressing them into high yielding cultivated varieties, will aid in minimizing losses caused by PHS. This will facilitate the development and implementation of high yield, stress-resistant mungbean varieties, ensuring consistent yields and increased profitability for mungbean farmers. Future research on mungbean improvement should prioritize multilocation field trials to assess the effectiveness of the identified PHS-tolerant genotypes across multiple environmental conditions. Furthermore, identifying genomic regions that govern PHS resistance will help to create varieties with stable, high yielding, and PHS and FSG resistance.

## Data Availability

The original contributions presented in the study are included in the article/**Supplementary Material**. Further inquiries can be directed to the corresponding authors.

## References

[B1] Ahmad S. Khulbe R. K. Roy D. (2014). Evaluation of mungbean germplasm for pre-harvest sprouting tolerance. Legume Res. 37, 259–263. doi: 10.5958/j.0976-0571.37.3.039

[B2] Ambhore A. M. Solanke A. P. Lipane R. R. Adagale R. V. Shamkuwar G. (2024). Insights from morpho-physiological traits imparting tolerance for pre-harvest sprouting of Mungbean genotypes. J. Adv. Biol. Biotechnol. 27, 434–440. doi: 10.9734/jabb/2024/v27i6902

[B3] Andrews C. H. (1982). Pre-harvest Environment: Weathering in Soybean Seed Quality and Stand Establishment (INTSOY) College of Agriculture, University of Illinois, Urbana, IL. 19–25.

[B4] Anupama S. Khulbe R. K. Panwar R. K. (2012). Evaluation of urdbean (*Vigna mungo*) germ plasm for pre-harvest sprouting tolerance. J. Food Legumes. 25, 183–186.

[B5] Anwar F. Latif S. Przybylski R. Sultana B. Ashraf M. (2007). Chemical composition and antioxidant activity of seeds of different cultivars of mung bean. J. Food Sci. 72, S503–S510. doi: 10.1111/j.1750-3841.2007.00462.x, PMID: 17995664

[B6] Bailey P. McKibbin R. Lenton J. Holdsworth M. J. Flintham J. E. Gale M. D. (1999). Genetic map locations for orthologous Vp1 genes in wheat and rice. Theor. Appl. Genet. 98, 281–284. doi: 10.1007/s001220051069

[B7] Baskin J. M. Baskin C. C. (2004). A classification system for seed dormancy. Seed Sci. Res. 14, 1–16. doi: 10.1079/SSR2003150

[B8] Black M. Bewley J. D. Halmer P. (2006). The encyclopedia of seeds: science, technology and uses (Wallingford, UK: CAB International). doi: 10.1079/9780851997230.0000

[B9] Cardon-Thomas D. K. Riviere T. Tieges Z. Greig C. A. (2017). Dietary protein in older adults: adequate daily intake but potential for improved distribution. Nutrients 9, 184. doi: 10.3390/nu9030184, PMID: 28241469 PMC5372847

[B10] Chauhan Y. S. Douglas C. Rachaputi R. C. N. Agius P. Martin W. King K.. (2010). Physiology of Mungbean and Development of the Mungbean Crop Model. In: 1st Australian Summer Grains Conference, 21st – 24th June 2010., Gold Coast, Department of Primary Industries, Queensland, Australia.

[B11] Cheng J. Schloerke B. Karambelkar B. Xie Y. (2025). leaflet: Create Interactive Web Maps with the JavaScript ‘Leaflet’ Library. R package version 2.2.2.9000. Available online at: https://rstudio.github.io/leaflet/ (Accessed July 7, 2025).

[B12] Cheralu C. Satyanarayana. A. Kulkarni. N. Jagdishwar K. Reddy M. S. (1999). Combining ability analysis for resistance to pre-harvest sprouting in mungbean [*Vigna radiata* (L.) Wilczek. Ind. J. Genet. 59, 465–472.

[B13] Dahiya P. K. Linnemann A. R. Van Boekel M. a. J.S. Khetarpaul N. Grewal R. B. Nout M. J. R. (2015). Mung Bean: Technological and nutritional potential. Crit. Rev. Food Sci. Nutr. 55, 670–688. doi: 10.1080/10408398.2012.671202, PMID: 24915360

[B14] Dhare S. L. Patil D. K. Gite V. K. Kalpande H. V. (2024). Assessment of genetic variances and effects for agronomic traits in mungbean (*Vigna radiata* (L.) Wilczek). Int. J. Advanced Biochem. Res. 8, 473–476. doi: 10.33545/26174693.2024.v8.i5f.1108

[B15] Dhare S. L. Patil D. K. Thakur N. R. Pawar G. S. (2025). Genetics of consumer-preferred morphological traits in contrasting parental crosses of mungbean (*Vigna radiata* (L.) Wilczek). Plant Breed. 0, 1–15. doi: 10.1111/pbr.70009

[B16] Dougherty R. W. Boerma H. R. (1984). Genotypic variation for resistance to pre harvest sprouting in soybean. Crop Sci. 24, 683–686. doi: 10.2135/cropsci1984.0011183X002400040014x

[B17] Durga K. K. Kumar S. S. (1997). Screening for pre-harvest sprouting in pulses. Legume Res. 20, 193–197.

[B18] Fang J. Chu C. (2008). Abscisic acid and the pre-harvest sprouting in cereals. Plant Signaling Behav. 3, 1046–1048. doi: 10.4161/psb.3.12.6606, PMID: 19513237 PMC2634458

[B19] Ganesan K. Xu B. (2018). A critical review on phytochemical profile and health promoting effects of mungbean (*Vigna radiata*). Food Sci. Hum. Wellness 7, 11–33. doi: 10.1016/j.fshw.2017.11.002

[B20] Gupta U. S. (2019). What’s New About Crop Plants: Novel Discoveries of the 21st Century (Boca Raton, FL, USA: CRC Press). doi: 10.1201/b10736

[B21] Gupta S. Aski M. Mishra G. P. Yadav P. S. Tripathi K. Lal S. K.. (2024). Genetic variation for tolerance to pre-harvest sprouting in mungbean (*Vigna radiata*) genotypes. PeerJ 12, e17609. doi: 10.7717/peerj.17609, PMID: 39071133 PMC11276771

[B22] Hammer K. (1984). The domestication syndrome. Kulturpflanze 32, 11–34. doi: 10.1007/BF02098682

[B23] Hampton J. G. Boelt B. Rolston M. P. Chastain T. G. (2013). Effects of elevated CO2 and temperature on seed quality. J. Agric. Sci. 151, 154–162. doi: 10.1017/S0021859612000263, PMID: 23495259 PMC3594839

[B24] Hijmans R. J. Barbosa M. Ghosh A. Mandel A. (2025). geodata: Download Geographic Data. R package version 0.6-3. Available online at: https://github.com/rspatial/geodata (Accessed July 7, 2025).

[B25] Hou D. Yousaf L. Xue Y. Hu J. Wu J. Hu X.. (2019). Mungbean (*Vigna radiata* L.): bioactive polyphenols, polysaccharides, peptides, and health benefits. Nutrients 11, 1238. doi: 10.3390/nu11061238, PMID: 31159173 PMC6627095

[B26] Humphry M. E. Lambrides C. J. Chapman S. C. Aitken E. Imrie B. C. Lawn R. J.. (2005). Relationships between hard-seededness and seed weight in mungbean (*Vigna radiata*) assessed by QTL analysis. Plant Breed. 124, 292–298. doi: 10.1111/j.1439-0523.2005.01084.x

[B27] Imrie B. C. (1983). “Response to selection for weathering resistance in mungbean,” in Proceedings of the Australian Plant Breeding Conference. Ed. Driscoll C. J.(Adelaide, Australia: Conference Organizing Committee), 348–350.

[B28] Imrie B. C. (1987) in Mungbean: Proceedings of the Second International Symposium, Bangkok, Thailand, November 16-20th, 130–135. Taipei, Taiwan: Asian Vegetable Research and Development Center.

[B29] Isemura T. Kaga A. Tabata S. Somta P. Srinives P. Shimizu T.. (2012). Construction of a genetic linkage map and genetic analysis of domestication related traits in mungbean (*Vigna radiata*). PloS One 7, e41304. doi: 10.1371/journal.pone.0041304, PMID: 22876284 PMC3410902

[B30] Jaiswal V. Mir R. R. Mohan A. Balyan H. S. Gupta P. K. (2012). Association mapping for pre-harvest sprouting tolerance in common wheat (*Triticum aestivum* L.). Euphytica 188, 89–102. doi: 10.1007/s10681-012-0713-1

[B31] Jayamani P. Muthiah A. R. Durairaj C. Pazhanivelan S. Kamalakanan A. Thiyagarajan K. (2015). Greengram Co 8 A high yielding, short duration variety with synchronized maturity. Madras Agric. J. 102, 1. doi: 10.29321/MAJ.10.001077

[B32] Jia Y. Barrero J. M. Wang J. Considine M. J. Nakamura S. Li C. (2024). Editorial: Seed dormancy, germination, and pre-harvest sprouting, volume II. Front. Plant Sci. 15. doi: 10.3389/fpls.2024.1399510, PMID: 38595760 PMC11002216

[B33] Kadam S. Jahagirdar J. Kalpande H. Kalyankar S. Deshmukh A. Thakur N.. (2022). Dormancy studies in *in-situ* germination in Mungbean (*Vigna radiata* L.). Pharma Innovation J. 11, 1413–1418.

[B34] Kadam S. R. Thakur N. R. Jahagirdar J. E. (2023). Study of correlation in mungbean (*Vigna radiata* L.) for seed yield and yield contributing traits. Seed Res. 51, 61–64.

[B35] Khare D. Raut N. D. Rao S. Lakhani J. P. (1995). Effect of seed size on germination and field emergence in soybean. Seed Res. 23, 75–79.

[B36] King R. W. Richards R. A. (1984). Water uptake in relation to pre-harvest sprouting damage in wheat: ear characteristics. Crop Pasture Sci. 35, 327–336. doi: 10.1071/AR9840337

[B37] Kolde R. (2018). pheatmap: Pretty Heatmaps. R package version 1.0.12. Available online at: https://github.com/raivokolde/pheatmap (Accessed July 7, 2025).

[B38] Koornneef M. Bentsink L. Hilhorst H. (2002). Seed dormancy and germination. Curr. Opin. Plant Biol. 5, 33–36. doi: 10.1016/S1369-5266(01)00219-9, PMID: 11788305

[B39] Krishnasamy V. Seshu D. V. (1990). Germination after accelerated aging and associated characters in rice varieties. Seed Sci. Technol. 18, 353–359.

[B40] Kueneman E. A. Dassou S. (1982). “Development of screening methods to breed soybean with resistance to field deterioration of seed,” in IITA Annual Report for 1981. 130–139. International Institute of Tropical Agriculture, Ibadan, Nigeria.

[B41] Kumar G. P. Pallavi M. Swapna N. Shahana E. Reddy G. E. Rakesh G. (2021). Genetic variability and correlation studies for pre-harvest sprouting tolerance and associated traits in soybean [*Glycine max* L. Merrill.]. Curr. J. App. Sci. Technol. 40 (4), 1–10. doi: 10.9734/cjast/2021/v40i431290

[B42] Kumari N. Malik D. (2024). Growth and instability in mungbean production in India. J. Food Legumes 37, 224–231. doi: 10.59797/jfl.v37.i2.199

[B43] Lamichaney A. Hazra K. K. Katiyar P. K. Parihar A. K. Gupta D. S. Kumar A.. (2023). Influence of seed and pod biophysical characters on pre-harvest sprouting tolerance in urdbean (*Vigna mungo* L.). Acta Physiologiae Plantarum 45, 48. doi: 10.1007/s11738-023-03533-8

[B44] Lamichaney A. Katiyar P. K. Laxmi V. Pratap A. (2017). Variation in pre−harvest sprouting tolerance and fresh seed germination in mungbean (*Vigna radiata* L.) genotypes. Plant Genet. Resour. 16, 437–445. doi: 10.1017/S1479262117000296

[B45] Lamichaney A. Katiyar P. K. Laxmi V and Pratap A. (2018). Variation in pre-harvest sprouting tolerance and fresh seed germination in mungbean (*Vigna radiata* L.) genotypes. Plant Genet. Resour. - Characterization Utilization 16, 437–445. doi: 10.1017/S1479262117000296

[B46] Lamichaney A. Pratap A. Katiyar P. K. Singh N. P. (2021). Genotypic variability studies and identification of pre-harvest sprouting tolerant wild *Vigna* . Indian J. Agric. Sci. 91, 335–339. doi: 10.56093/ijas.v91i3.112439

[B47] Laosatit K. Amkul K. Yimram T. Chen J. Lin Y. Yuan X.. (2022). A class II KNOX gene, *KNAT7-1*, regulates physical seed dormancy in mungbean [*Vigna radiata* (L.) Wilczek. Front. Plant Sci. 13. doi: 10.3389/fpls.2022.852373, PMID: 35371162 PMC8965505

[B48] Lawn R. J. Williams R. W. Imrie B. C. (1986). “Wild germplasm as a source of tolerance to environmental stresses in mungbean,” in Food Legume Improvement for Asian Farming Systems - Proceedings of an international workshop held in Khon Kaen, Thailand, 1–5 September 1986. Eds. Wallis E. S. Byth D. E. Ramsay Ware Printing, Melbourne.

[B49] Lawn R. J. Williams R. W. Imrie B. C. (1988). “Potential of wild germplasm as a source of tolerance to environmental stresses in mungbean in Mungbean,” in Proceedings of the Second International Symposium. Eds. Shanmugasundaram S. McLean B. T. (Asian Vegetable Research and Development Centre, Shanhua), 136–145.

[B50] Li L. Zhang Y. Zhang Y. Li M. Xu D. Tian X.. (2021). Genome-wide linkage mapping for pre-harvest sprouting resistance in wheat using 15K single-nucleotide polymorphism arrays. Front. Plant Sci. 12. doi: 10.3389/fpls.2021.749206, PMID: 34721477 PMC8551680

[B51] Lin M. Zhang D. Liu S. Zhang G. Yu J. Fritz A. K.. (2016). Genome-wide association analysis on pre-harvest sprouting resistance and grain color in U.S. winter wheat. BMC Genomics 17, 794. doi: 10.1186/s12864-016-3148-6, PMID: 27729004 PMC5059910

[B52] Liu S. Sehgal S. K. Li J. Lin M. Trick H. N. Yu J.. (2013). Cloning and characterization of a critical regulator for pre-harvest sprouting in wheat. Genetics 195, 263–273. doi: 10.1534/genetics.113.152330, PMID: 23821595 PMC3761307

[B53] Maity A. Vijay D. Mukherjee A. Lamichaney A. (2016). Potential impacts of climate change on quality seed production: a perspective of hill agriculture. In Bisht J. Meena V. Mishra P. Pattanayak A. (eds) Conservation Agriculture. Springer, Singapore.

[B54] Mares D. J. Mrva K. (2014). Wheat grain pre-harvest sprouting and late maturity alpha-amylase. Planta 240, 1167–1178. doi: 10.1007/s00425-014-2172-5, PMID: 25257145

[B55] Mishra G. P. Dikshit H. K. Tripathi K. Aski M. S. Pratap A. Dasgupta U.. (2022). “Mungbean breeding,” in Fundamentals of Field Crop Breeding. Eds. Yadava D. K. Dikshit H. K. Mishra G. P. Tripathi S. (Springer, Singapore).

[B56] Miyagi M. Humphry M. E. Ma Z. Y. Lambrides C. J. Bateson M. Liu C. J.. (2004). Construction of bacterial artificial chromosome libraries and their application in developing PCR-based markers closely linked to a major locus conditioning bruchid resistance in mungbean (*Vigna radiata* L. Wilczek). Theor. Appl. Genet. 110, 151–156. doi: 10.1007/s00122-004-1821-7, PMID: 15490104

[B57] Mogali S. Patil N. K. B. Ranjita H. Balol G. Jaggal L. (2023). Development of mungbean genotypes for shattering tolerance and correlation analysis with biochemical and morphological factors governing pre harvest sprouting. Legume Res. 48, 1434-1441. doi: 10.18805/LR-5089

[B58] Mogotsi K. K. (2006). “ *Vigna radiata* (L.) Wilczek,” in PROTA 1: Cereals and Pulses/Cereals et legumes secs. Eds. Brink M. Belay G. (PROTA, Wageningen, Netherlands).

[B59] Nair R. M. Yang R. Y. Easdown W. J. Thavarajah D. Thavarajah P. Hughes J. D.. (2013). Biofortification of mungbean (*Vigna radiata*) as a whole food to enhance human health. J. Sci. Food Agric. Jun 93, 1805–1813. doi: 10.1002/jsfa.6110, PMID: 23426879

[B60] Nalajala S. Muniyandi S. J. Manjunath P. (2023). Principal component analysis of quantitative and qualitative traits in sixty mung bean (*Vigna radiata* L. Wilczek) genotypes. Int. J. Bio-resource Stress Manage. 14, 643–651. doi: 10.23910/1.2023.3354

[B61] Nautiyal P. C. Bandyopadhyay A. Zala P. V. (2001). *In situ* sprouting and regulation of fresh seed dormancy in Spanish type groundnut (*Arachis hypogaea* L.). Field Crops Res. 70, 233–241. doi: 10.1016/S0378-4290(01)00143-5

[B62] Odong T. L. Van Heerwaarden J. Van Hintum T. J. L. Van Eeuwijk F. A. Jansen J. (2013). Improving hierarchical clustering of genotypic data via principal component analysis. Crop Sci. 53, 1546–1554. doi: 10.2135/cropsci2012.04.0215

[B63] Pal S. S. Singh J. J. Singh I. (2000). Transfer of YMV resistance in cultivar SML32 of *Vigna radiata* from other related *Vigna* species. Plant Dis. Res. 15, 67–69.

[B64] Palaniyappan S. Arunachalam P. Banumathy S. Muthuramu S. (2024). Principal component analysis (PCA) as a genetic diversity tool to understand the variation of rice mutant culture. Elec. J. Pl. Breed. 15, 979-985. doi: 10.37992/2024.1504.113

[B65] Panse V. G. Sukhatme P. V. (1967). Statistical method for agri cultural workers. 4th edn (New Delhi: Indian Council of Agricultural Research).

[B66] Paul D. Chakrabarty S. K. Nain L. Maity A. (2025). Studies on key seed physico-chemical factors and climatic variables regulating hard-seededness in green gram (*Vigna radiata* L.) genotypes. Crop Pasture Sci. 76. doi: 10.1071/cp24259

[B67] Pearson K. (1920). Notes on the history of correlation. Biometrika 13, 25–45. doi: 10.1093/biomet/13.1-2.25

[B68] Pebesma E. Bivand R. (2023). Spatial Data Science: With applications in R (New York: Chapman and Hall/CRC). doi: 10.1201/9780429459016

[B69] Posit Team (2025). RStudio: Integrated Development Environment for R (Boston, MA: Posit Software, PBC). Available online at: http://www.posit.co/ (Accessed July 7, 2025).

[B70] Pramod P. J. S. Hari Satyanarayana N. Sateesh Babu J. Jaya Lalitha and K. Roja. V. (2024). Genetic variability and association studies for yield and pre-harvest sprouting traits in greengram [*Vigna radiata* L. Wilczek. Electronic J. Plant Breed. 15, 952–961. doi: 10.37992/2024.1504.111

[B71] Pratap A. Das A. Agrawal S. K. Gupta S. (2021). Current perspectives on introgression breeding in food legumes. Front. Plant Sci. 11. doi: 10.3389/fpls.2020.589189, PMID: 33552095 PMC7858677

[B72] Pratap A. Gupta S. Basu S. Tomar R. Dubey S. Rathore M.. (2019). “Towards development of climate-smart mungbean: challenges and opportunities,” in Genomic Designing of Climate Smart Pulse Crops. Ed. Kole C. (Springer Nature, New York). doi: 10.1007/978-3-319-96932-9_5

[B73] Rao N. K. Hanson J. Dulloo M. E. Ghosh K. Nowell D. Larinde M.. (2006). Manual of seed handling in genebanks. Handbooks for Genebanks No. 8. Bioversity International, Rome, Italy.

[B74] Rao P. S. Madhulety T. Y. Ankaiah R. Voleti S. R. (2023). Morpho-physiological variation of pre-harvest tolerance to simulated rain in mungbean (*Vigna radiata*). Indian J. Agric. Sci. 93, 269–273. doi: 10.56093/ijas.v93i3.128735

[B75] Rao K. L. N. Rao C. M. Rao Y. K. (2007). “Evaluation of greengram germplasm for tolerance to pre-harvest sprouting,” in Environmental Protection. Eds. Kumar A. Daya N. S. (Publishing House, Delhi), 51–54.

[B76] Rashid M. Hampton J. G. Rolston M. P. Khan K. M. Saville D. J. (2018). Heat stress during seed development affects forage brassica (*Brassica napus* L.) seed quality. J. Agron. Crop Sci. 204, 147–157. doi: 10.1111/jac.12251

[B77] Reddy A. D. Harini A. S. Kumar S. S. Anjali C. Raghavendra P. (2025). Variation for pre-harvest sprouting resistance in mungbean (*Vigna radiata* (L.). J. Exp. Agric. Int. 47, 322–333. doi: 10.9734/jeai/2025/v47i63492

[B78] Rodriguez M. V. Arata G. J. Díaz S. M. Rentería S. Benech-Arnold R. L. (2021). Phenotyping for resistance to pre-harvest sprouting in grain sorghum. Seed Sci. Res. 31, 1–10. doi: 10.1017/S0960258521000076

[B79] Sathuri P. Kyada A. D. Kale B. H. Patel G. M. Modha K. G. Chauhan D. A.. (2023). Genetic grouping of selected RILs for yield and attributing traits in determinate type of Indian bean. Electronic J. Plant Breed. 14, 1206–1214. doi: 10.37992/2023.1403.146

[B80] Schafleitner R. Huang S. M. Chu S. H. Yen J. Y. Lin C. Y. Yan M. R.. (2016). Identification of single nucleotide polymorphism markers associated with resistance to bruchids (*Callosobruchus spp*.) in wild mungbean (*Vigna radiata* var. *sublobata*) and cultivated *V. radiata* through genotyping by sequencing and quantitative trait locus analysis. BMC Plant Biol. 16, 159. doi: 10.1186/s12870-016-0847-8, PMID: 27422285 PMC4946214

[B81] Sharma S. Mittal R. Sood V. Chaudhary H. (2022). Development of interspecific hybrids between urdbean & mungbean. Environ. Conserv. J. 23, 315–321. doi: 10.36953/ECJ.021892-2171

[B82] Sievert C. (2020). Interactive Web-Based Data Visualization with R, plotly, and shiny (Chapman and Hall/CRC). Available online at: https://plotly-r.com (Accessed July 7, 2025).

[B83] Simsek S. Ohm J. B. Lu H. Rugg M. Berzonsky W. Alamri M. S.. (2014). Effect of pre-harvest sprouting on physicochemical properties of starch in wheat. Foods 3, 194–207. doi: 10.3390/foods3020194, PMID: 28234313 PMC5302366

[B84] Singh P. Chourasiya V. K. Verma P. (2017). Screening of Mungbean (*Vigna radiata*) germplasm against precocious germination susptability. Int. J. Pure App. Biosci. 5, 1010–1014. doi: 10.18782/2320-7051.4010

[B85] Singh D. P. Hooda M. S. Bonner F. T. (1991). An evaluation of scarification methods for seeds of two leguminous trees. New Forests 5, 139–145. doi: 10.1007/BF00029304

[B86] Singh P. Jain P. K. Tiwari A. (2020). Principal component analysis approach for yield attributing traits in chilli (*Capsicum annum* L.) genotypes. Chem. Sci. Rev. Lett. 9, 87–91. doi: 10.37273/chesci.cs232050121

[B87] Singh A. Khulbe R. K. Panwar R. K. (2012). Evaluation of black gram (*Vigna mungo*) germplasm for pre-harvesting sprouting tolerance. J. Food. Legumes. 25, 183–186.

[B88] Singh D. P. Sharma B. L. Dwivedi S. (1983). Inheritance of hard seeds in inter-specific crosses of Mungbean. Indian J. Genet. 43, 378–379.

[B89] Singh P. Singh S. Jadhav C. B. S. Bhargava M. K. Puneet K. (2021). Combining ability and gene action for seed yield and its component traits in green gram (*Vigna radiata* L.). Ann. Plant Soil Res. 23, 116–122. doi: 10.47815/apsr.2021.10041

[B90] Smýkal P. Nelson M. N. Berger J. D. Von Wettberg E. J. (2018). The impact of genetic changes during crop domestication. Agronomy 8, 119. doi: 10.3390/agronomy8070119

[B91] Somta P. Laosatit K. Yuan X. Chen X. (2022). Thirty years of mungbean genome research: Where do we stand and what have we learned? Front. Plant Sci. 13. doi: 10.3389/fpls.2022.944721, PMID: 35909762 PMC9335052

[B92] Sunayana R. Ramendra N. S. Raj N. S. D. (2013). Variation in seed dormancy and α-amylase activity in Indian rice (*Oryza sativa*) accessions. Indian J. Agric. Sci. 83, 56–62.

[B93] Tai L. Wang H. J. Xu X. J. Sun W. H. Ju L. Liu W. T.. (2021). Pre-harvest sprouting in cereals: genetic and biochemical mechanisms. J. Exp. Bot. 72, 2857–2876. doi: 10.1093/jxb/erab024, PMID: 33471899

[B94] Thakur N. R. Ingle K. P. Sargar P. R. Baraskar S. S. Kasanaboina K. Awio B.. (2024). “Sustainable utilization of wild germplasm resources,” in Sustainable Utilization and Conservation of Plant Genetic Diversity. Ed. Al-Khayri J. M. (Springer Nature Singapore), 551–557. doi: 10.1007/978-981-99-5245-8_16

[B95] Tomooka N. Lairungruang C. Nakeeraks P. Egawa Y. Thavarasook C. (1992). Development of bruchid resistant mungbean using wild mungbean germplasm in Thailand. Plant Breed. 109, 60–66. doi: 10.1111/j.1439-0523.1992.tb00151.x

[B96] Venable D. L. (2007). Bet hedging in a guild of desert annuals. Ecology 88, 1086 1090. doi: 10.1890/06-1495, PMID: 17536393

[B97] Verma J. Gore P. G. Kumari J. Wankhede D. P. Jacob S. R. Thirumani Venkatesh A. K.. (2024). Exploring genetic diversity in black gram (*Vigna mungo* (L.) Hepper) for pre-harvest sprouting tolerance. Agronomy 14, 197. doi: 10.3390/agronomy14010197

[B98] Vetch J. M. Stougaard R. N. Martin J. M. Giroux M. J. (2019). Revealing the genetic mechanisms of pre-harvest sprouting in hexaploid wheat (*Triticum aestivum* L.). Plant Sci. 281, 180–185. doi: 10.1016/j.plantsci.2019.01.004, PMID: 30824050

[B99] Vijay L. Gupta S. (2008). “Pre-harvest sprouting tolerance in mungbean,” in Pulses News Letter, vol. 19. (Indian Institute of Pulses Research, Kanpur, India), 4–5.

[B100] Wickham H. (2016). ggplot2: Elegant Graphics for Data Analysis (Springer-Verlag New York). Available online at: https://ggplot2.tidyverse.org (Accessed July 7, 2025).

[B101] William R. W. (1984) in Australian Seed Research Conference, Lawes. 191–197. Queensland Department of Primary Industries, Brisbane. Queensland, Australia.

